# Activation of the integrative and conjugative element Tn*916* causes growth arrest and death of host bacteria

**DOI:** 10.1371/journal.pgen.1010467

**Published:** 2022-10-24

**Authors:** Emily L. Bean, Lisa K. McLellan, Alan D. Grossman

**Affiliations:** Department of Biology Massachusetts, Institute of Technology Cambridge, Massachusetts, United States of America; Institut Cochin, FRANCE

## Abstract

Integrative and conjugative elements (ICEs) serve as major drivers of bacterial evolution. These elements often confer some benefit to host cells, including antibiotic resistance, metabolic capabilities, or pathogenic determinants. ICEs can also have negative effects on host cells. Here, we investigated the effects of the ICE (conjugative transposon) Tn*916* on host cells. Because Tn*916* is active in a relatively small subpopulation of host cells, we developed a fluorescent reporter system for monitoring activation of Tn*916* in single cells. Using this reporter, we found that cell division was arrested in cells of *Bacillus subtilis* and *Enterococcus faecalis* (a natural host for Tn*916*) that contained an activated (excised) Tn*916*. Furthermore, most of the cells with the activated Tn*916* subsequently died. We also observed these phenotypes on the population level in *B*. *subtilis* utilizing a modified version of Tn*916* that can be activated in the majority of cells. We identified two genes (*orf17* and *orf16*) in Tn*916* that were sufficient to cause growth defects in *B*. *subtilis* and identified a single gene, *yqaR*, that is in a defective phage (*skin*) in the *B*. *subtilis* chromosome that was required for this phenotype. These three genes were only partially responsible for the growth defect caused by Tn*916*, indicating that Tn*916* possesses multiple mechanisms to affect growth and viability of host cells. These results highlight the complex relationships that conjugative elements have with their host cells and the interplay between mobile genetic elements.

## Author summary

Horizontal gene transfer, often mediated by mobile genetic elements, facilitates bacterial evolution. Integrative and conjugative elements (ICEs) are mobile genetic elements that encode machinery to transfer the element from a donor bacterium to a neighboring cell, thereby generating a new bacterial host. ICEs are the most prevalent type of conjugative element and confer novel traits, including antibiotic resistances and symbiotic, pathogenic, or metabolic capabilities. Early-discovered ICEs were identified by the benefits they provide to their host cells. Tn*916*, the first-described ICE, was identified due to its ability to spread tetracycline resistance in clinical isolates of *Enterococcus*. However, conjugative elements can also incur a cost to their hosts. Here, we report that the activated form of Tn*916* causes growth arrest and cell death in two bacterial hosts: *Bacillus subtilis*, which has been used for many studies of the element; and *Enterococcus faecalis*, a natural host of the element. We demonstrate that these deleterious effects in *B*. *subtilis* are due, at least in part, to interactions between components of the Tn*916* conjugation machinery and a co-resident prophage-like element. Our findings highlight the complex nature of interactions that can occur between an integrative and conjugative element, its host, and other horizontally acquired elements.

## Introduction

Integrative and conjugative elements (ICEs), also called conjugative transposons, are mobile genetic elements that contribute to bacterial evolution. Typically, an ICE resides integrated in the chromosome of a bacterial host. Either stochastically or in response to a signal, an ICE can excise to form an extrachromosomal circle. ICE-encoded conjugation machinery (a type IV secretion system, T4SS) can transfer the ICE into a recipient cell in a contact-dependent manner [1–5].

Conjugative elements often carry genes that confer phenotypes to host cells, including antibiotic resistances, pathogenic or symbiotic abilities, and various metabolic capabilities. Conjugative elements were frequently identified based on the phenotypes that they confer to bacterial hosts {reviewed in: [3]}. Advantageous phenotypes conferred by ICEs likely mitigate potential costs of maintaining these elements.

Conjugative elements can also have more complex relationships with their host cells. Some elements encode functions that manipulate host development, growth, and viability (for examples see [6–9]). Excessive mating events can be detrimental to host viability [10,11]. Additionally, interactions between conjugative elements and other horizontally-acquired elements, including phages, can impact a host cell. For instance, pili that are part of some conjugation systems can be targeted by male-specific phages [12–14]. Some phages can prevent conjugation events from occurring [15–17], and at least one ICE can prevent growth of a specific phage via an abortive infection mechanism [18].

Here, we present evidence that the ICE Tn*916* possesses a previously unknown ability to cause a growth arrest and kill its host cell. Tn*916* was the first described ICE and was identified based on its ability to spread tetracycline resistance between two strains of *Enterococcus faecalis* [19,20]. Tn*916* and its relatives have since been found in other Gram-positive bacteria including *Streptococcus*, *Staphylococcus*, and *Clostridium* species [4,21–26], and it is functional in *Bacillus subtilis* [27–32]. Tn*916* is regulated, at least in part, by a transcriptional attenuation mechanism that is relieved in the presence of tetracycline or other antibiotics that inhibit translation [4,33–35]. These drugs stimulate excision and transfer of Tn*916* [32,33,36,37]. However, Tn*916* only activates and excises in ~0.1–3% of cells in a population [32,33,38–40]. Therefore, any effects Tn*916* activation has on the host cell would be masked in population-level analyses.

To study the effects of Tn*916* gene activation on the population level in *B*. *subtilis* host cells, we used a hybrid conjugative element that contains the regulatory and recombination genes from a heterologous element and the DNA processing and conjugation genes from Tn*916* [41]. Using this hybrid element, we identified two Tn*916* genes that are sufficient to cause *B*. *subtilis* host cells to stop growing. We also identified a gene in the defective phage *skin* that was required for the growth defects caused by the two Tn*916* genes.

We also analyzed the effects of Tn*916* on cell growth in single cells using a fluorescent reporter to monitor activation of Tn*916*. We found that cell growth and division was inhibited in cells with an activated (excised) Tn*916*. Furthermore, most of these cells died. When activated in its natural host, *E*. *faecalis*, Tn*916* also caused growth arrest and cell death. We suggest that these growth defects may be a common feature across other bacterial hosts of Tn*916* and Tn*916*-like elements. Our results also indicate that the growth arrest likely functions to limit the spread of the element.

## Results

### Increased activation of Tn*916* genes causes defects in cell growth and viability

**Fig 1:**
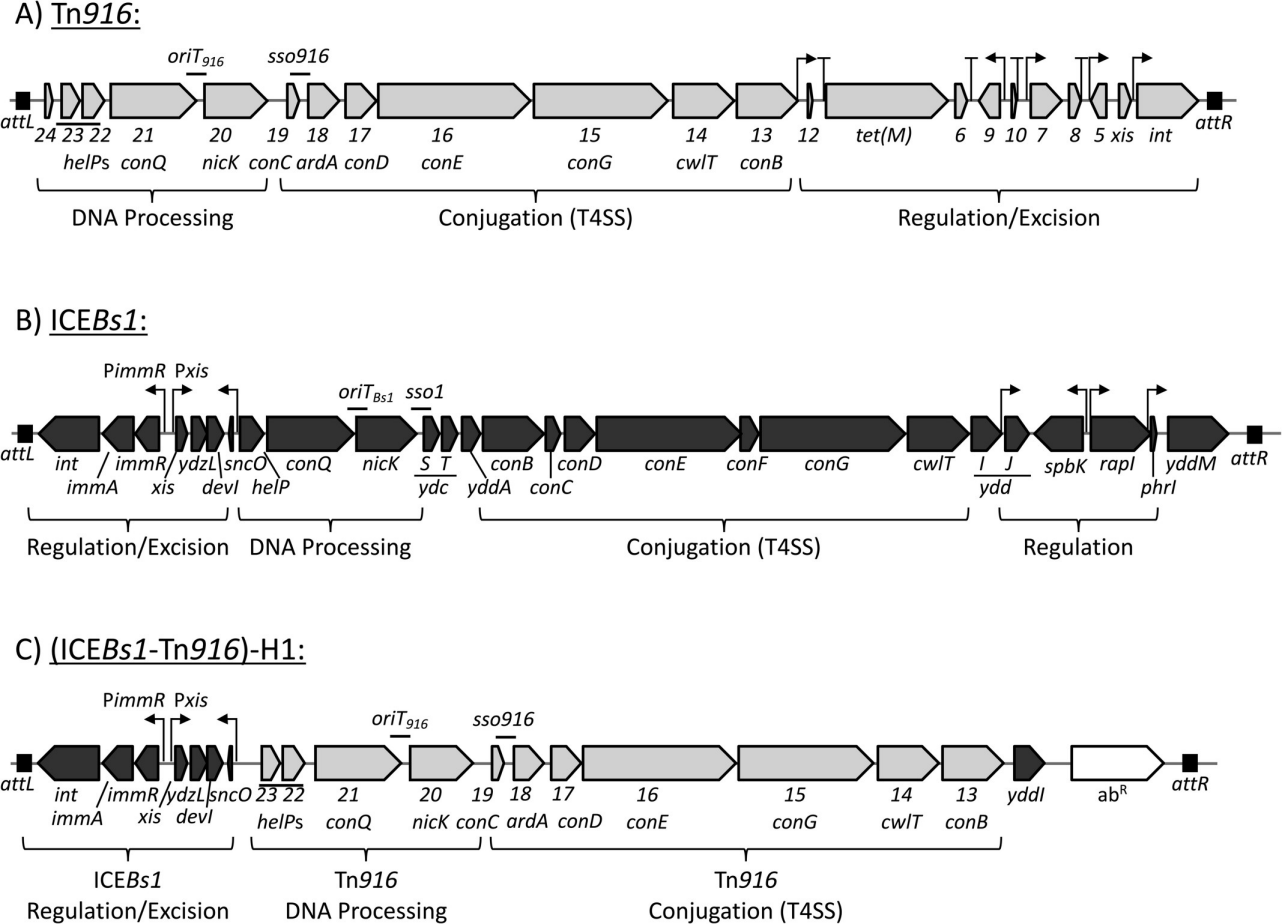
Genetic maps of Tn*916*, ICE*Bs1*, and (ICE*Bs1*-Tn*916*)-H1. Maps of the conjugative elements used in these studies are shown: **A)** Tn*916*, **B)** ICE*Bs1*, and **C)** (ICE*Bs1*-Tn*916*)-H1 (or ICE-H1, for short). Open reading frames are indicated by horizontal boxes with arrows at the end (gray for Tn*916*, black for ICE*Bs1*). Tn*916* gene names are abbreviated to include only the number designation from the name (i.e., “*orf23”* is written as “*23*”), and, when appropriate, the homologous ICE*Bs1* gene is written below. ICE-H1 contains a combination of Tn*916* and ICE*Bs1* genes, as previously described [41]. Functional modules are indicated by brackets below each map. Black boxes indicate attachment sites *attL* and *attR* at the ends of each element. In *B*. *subtilis*, Tn*916* is integrated between *yufK* and *yufL*, unless otherwise indicated. ICE*Bs1* and ICE-H1 are integrated at *trnS*-*leu2*. Some of the promoters are indicated by bent arrows and some transcription terminators (in Tn*916*) are indicated by “T” shapes. ICE-H1-Δ*attR Δorf20* {ICE-H1-*ΔattR* (Rep-)} is essentially the same as ICE-H1 (panel C) with a deletion of *attR* (right end, from ICE*Bs1*) and *orf20* (encoding the relaxase needed for nicking at *oriT*, conjugative transfer, and autonomous rolling circle replication). Previously determined origins of transfer (*oriT*) and single strand origins of replication (*sso*) are indicated by a “-”above the genetic map [32,84,92,93]. To induce activation of ICE*Bs1* and ICE-H1 and their derivatives, the activator RapI was overproduced from Pxyl-*rapI* (located at an ectopic locus on the chromosome; see Table 2). Active RapI causes the anti-repressor and protease ImmA to cleave the ICE*Bs1* repressor ImmR, thereby causing derepression of transcription from the promoter Pxis [42,43,83]. This figure is adapted from [41].

Tn*916*, like many conjugative elements, only becomes active and excises from the genome in a small portion (~0.1–3%) of the cells in a population [32,33,38–40]. In previous work, we created hybrid ICEs that contained the DNA processing and conjugation functions of Tn*916* (Fig 1A) and the efficient regulatory and recombination (integration and excision) systems encoded by ICE*Bs1* (Fig 1B) [41]. We refer to this hybrid element as (ICE*Bs1*-Tn*916*)-H1, or ICE-H1 for short (Fig 1C). ICE*Bs1* and ICE-H1 can be activated in ~25–90% of the cells in a population by overproduction of the ICE*Bs1-*encoded activator protein RapI. The presence of active RapI in the cell stimulates cleavage of the ICE*Bs1* repressor ImmR by the anti-repressor and protease ImmA. This causes derepression of transcription from the major promoter in ICE*Bs1*, Pxis, which drives transcription of most of the genes in the element, including those needed for excision, autonomous replication, and conjugation [41–44]. In the hybrid ICE-H1, Pxis drives transcription of the genes from Tn*916* that were inserted in place of the ICE*Bs1* genes and are needed for autonomous replication and conjugation (Fig 1). The ability to activate Tn*916* genes needed for DNA processing and conjugation in a large proportion of cells enables population-level analyses of effects of these genes on host cells.

We monitored the growth and viability of host cells that were growing in defined minimal medium under activating and non-activating conditions: xylose was added to induce expression of *rapI* (from Pxyl-*rapI*, located at the non-essential *amyE* locus) and activation of either ICE-H1 (ELC1214) or ICE*Bs1* (MMB970); tetracycline (2.5 μg/ml) was added to stimulate Tn*916* activation. ICE excision events were monitored via qPCR by quantifying the amounts of empty ICE attachment sites in the chromosome relative to a nearby chromosomal locus (Materials and Methods). By two hours after induction, excision had occurred in ~69%, ~95%, and 0.07% of cells containing ICE-H1, ICE*Bs1*, and Tn*916*, respectively.

**Fig 2:**
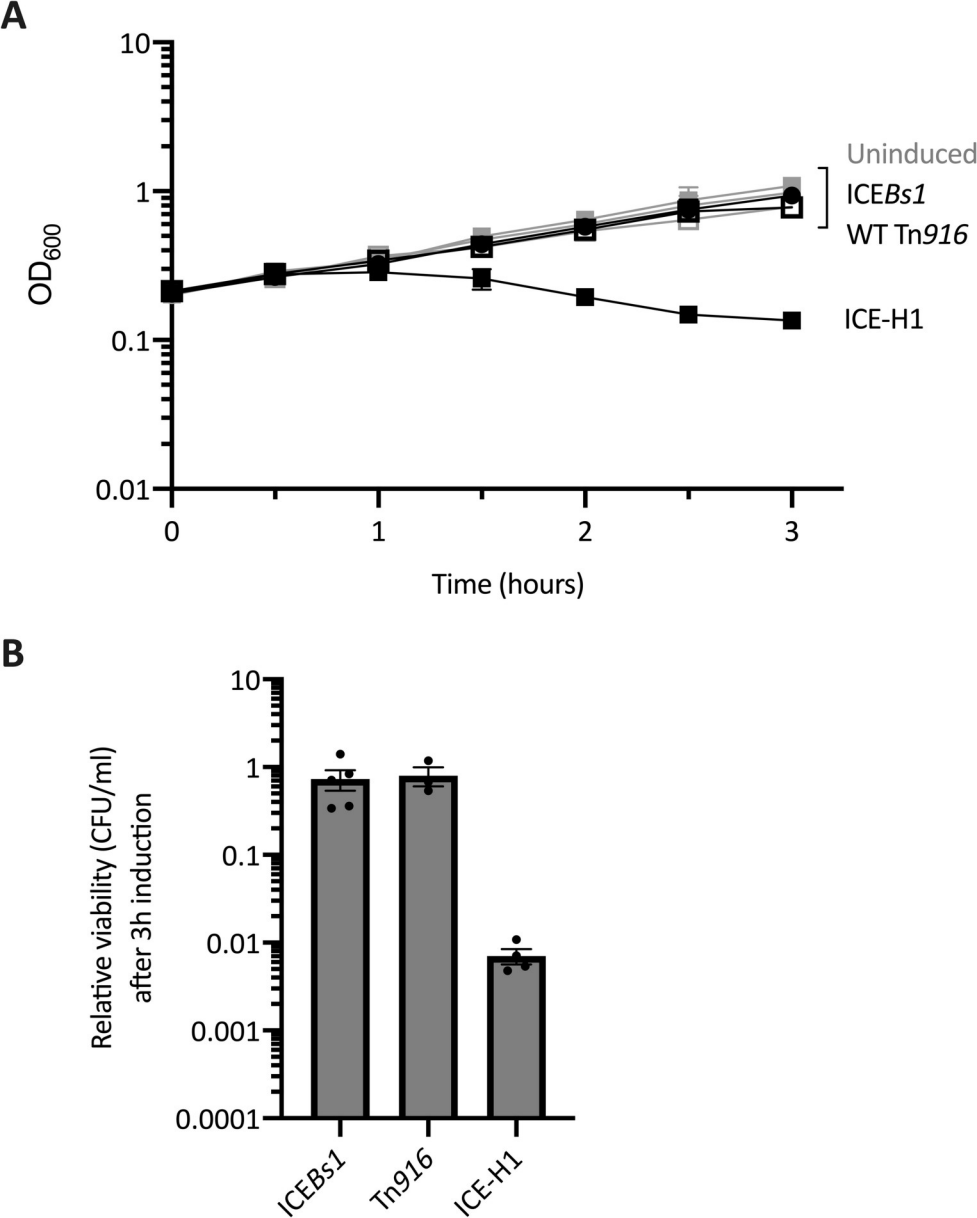
Activation of ICE-H1 causes growth arrest and cell death. Strains containing ICE*Bs1* (MMB970), Tn*916* (CMJ253) or ICE-H1 (ELC1214) were grown in defined minimal medium with arabinose to early exponential phase. Cultures were split into two at an OD of ~0.2 (indicated as time = 0 hours) and the appropriate inducer was added (1% xylose to stimulate *rapI* expression, or +2.5 μg/ml tetracycline to stimulate Tn*916* activation) to one part and the second part was left without induction. Data from four or more experiments (except for the growth curves for ICE*Bs1* and ICE-H1, time points 0.5, 1.5. and 2.5 h which were from two independent experiments) are presented as averages (**A**) or individual data points (**B**), and error bars represent standard error of the mean. **A)** Growth was monitored by OD_600_ for three hours. Gray lines indicate growth of uninduced cultures. Black lines indicate growth of the induced cultures; ICE*Bs1* (filled circles); Tn*916* (open squares); ICE-H1 (filled squares). Error bars could not always be depicted due to the size of each data point. **B)** The relative colony forming units per ml (CFUs/ml) of cultures after three hours of element activation was calculated as the number of CFUs formed by the induced culture, divided by that from the uninduced culture (a value of “1” indicates there is no change in CFUs with induction). Significant differences based on P < 0.05 in unpaired two-tailed T-tests include comparisons between ICE-H1 and each of the other elements.

Approximately one hour after induction of ICE-H1, growth of the culture stopped as measured by optical density (Fig 2A). The optical density of the culture then declined (Fig 2A), indicating that cell lysis was likely occurring. Indeed, after three hours, there was an approximately 100-fold drop in viable cells in the culture in which ICE-H1 was activated relative to the uninduced culture, as measured by colony forming units (CFUs) (Fig 2B). The combination of the drop in optical density and the drop in CFUs indicated that the majority of cells in the culture were lysing.

In contrast, cells in which ICE*Bs1* had been induced continued to grow, plateaued at a relatively normal optical density (Fig 2A), and there was no evidence of a large drop in cell viability (Fig 2B). Cultures of Tn*916*-containing cells also grew normally (Fig 2A) and there was no apparent drop in cell viability (Fig 2B). Of course, even if all of the cells in which Tn*916* had become activated (~0.1%) had lost viability, we would not detect this on a population level with the assays used (Fig 2). Based on the effects of ICE-H1 and ICE*Bs1* on cell growth and viability, we infer that the defects caused by ICE-H1 were either due to increased expression of genes from Tn*916* or the absence of a protective gene(s) from ICE*Bs1* in the ICE*Bs1*-Tn*916* hybrid.

**Fig 3:**
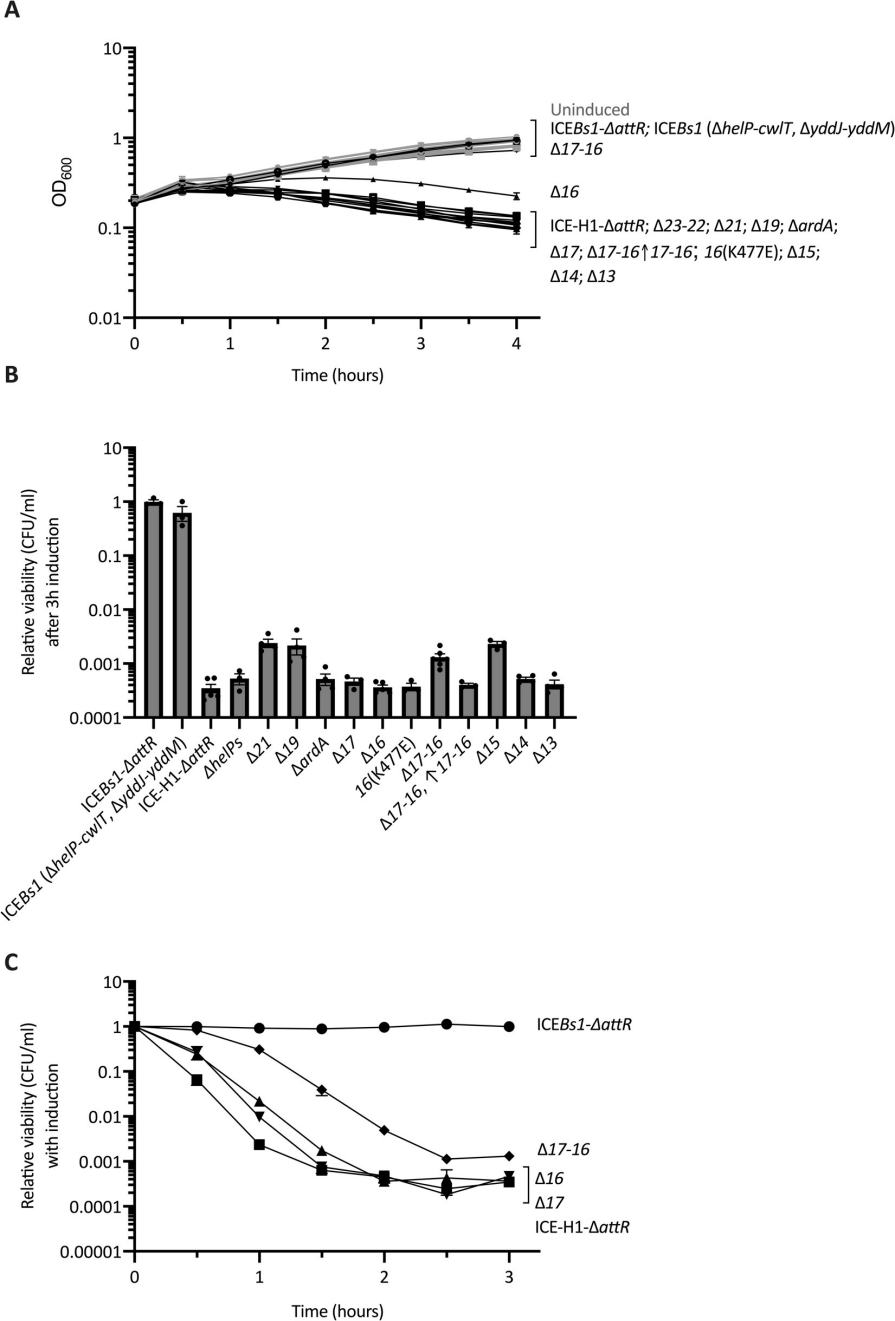
*orf16* and *orf17* are involved in the growth arrest caused by ICE-H1. Strains containing ICE*Bs1*-Δ*attR* (Rep- due to Δ*nicK*) (closed circles, ELC1095), ICE*Bs1* (Δ*helP*-*cwlT*, Δ*yddJ*-*yddM*) (open circles, ELC1226), or ICE-H1-Δ*attR* (Rep- due to *Δorf20*) (closed squares, ELC1076) with the indicated deletion(s) are indicated in the figure. Deletions are indicated by gene name or number and include: Δ*orf23*-*22* (open squares, ELC1945), Δ*orf21* (closed hexagons, ELC1916), Δ*orf19* (open hexagons, ELC1915), Δ*ardA* (stars, ELC1707), Δ*orf17* (closed downward triangle, ELC1419), Δ*orf16* (closed upward triangles, ELC1420), Δ*orf17*-*16* (closed diamonds, ELC1942), Δ*orf17*-*orf16* (with *lacA*::P*xis*-*orf17*-*orf16*; plus signs, ELC1550), *orf16*(*K477E*) (asterisks, ELC1899), Δ*orf15* (open downward triangles ELC1418), Δ*orf14* (open upward triangles, ELC1708), and *Δorf13* (open diamonds, ELC1705). Strains were grown in minimal arabinose medium to early exponential phase. At time = 0 hours, when cultures were at an OD_600_ ~0.2, cultures were split into inducing (+1% xylose to stimulate *rapI* expression) and non-inducing conditions. Data from three or more experiments are presented as averages (**A, C**) or individual data points (**B**), and error bars represent standard error of the mean. **A)** Growth was monitored by OD_600_ for three hours. Black lines indicate growth of the indicated induced cultures; gray lines (difficult to see as they are clustered in the set of strains at the top of the graph) indicate growth of uninduced cultures. Error bars could not always be depicted due to the size of each data point. Growth of the strains clustered at the top were virtually indistinguishable from each other. Growth of the strains clustered at the bottom were virtually indistinguishable from each other, but clearly different from those at the top. In between was the strain with *Δorf16*, which was consistently different from all the others. **B)** The relative CFUs/ml of cultures after three hours of element activation was calculated as the number of CFUs formed by the induced culture, divided by that from the uninduced culture (a value of “1” indicates there is no change in CFUs with induction). Significant differences based on P < 0.05 in unpaired two-tailed T-tests include comparisons between: ICE-H1-*ΔattR* (Rep-) and ICE*Bs1*-*ΔattR* (Rep-); ICE-H1-*ΔattR* (Rep-) and ICE-H1-Δ*attR* (Rep-) containing either Δ*orf21*, Δ*orf19*, Δ*orf17*-16, or Δ*orf15*. **C)** The relative CFUs/ml of cultures were evaluated every 30 minutes for three hours post-induction and were calculated as the number of CFUs formed by the induced culture, divided by that from the uninduced culture (a value of “1” indicates there is no change in CFUs with induction). Data for the 3 hr time point are the same as in the bar graph in panel B. Error bars could not always be depicted due to the size of each data point.

Additional experiments demonstrated that the growth arrest and cell death caused by induction of ICE-H1 were not due to loss of some putative protective gene(s) in ICE*Bs1*. We used a mutant of ICE*Bs1* that contained only the ICE*Bs1* genes present in ICE-H1. That is, the mutant {ICE*Bs1* (Δ*helP*-Δ*cwlT*, Δ*yddJ*-*yddM*); strain ELC1226} was missing all the ICE*Bs1* genes that were also missing in ICE-H1, and also did not contain any genes from Tn*916*. Activation of this element did not cause a growth arrest or drop in viability (Fig 3A and 3B), indicating that the growth arrest was not due to the absence of one or more postulated protective gene from ICE*Bs1*. Together, our results indicate that, when expressed, one or more genes from Tn*916* that are present in ICE-H1 cause growth arrest and cell death.

### Neither excision nor replication were required for the growth arrest and cell death caused by ICE-H1

We wished to determine which of the genes from Tn*916* that are present in ICE-H1 caused cell death. To simplify the analysis, we used an ICE-H1 mutant that was unable to excise from the host chromosome, and unable to replicate, even after activation of element gene expression. We made an excision-defective mutant of ICE-H1 by deleting the “right” attachment site (*attR*) (Fig 1) that is necessary for element excision. The gene encoding the relaxase (*nicK* from ICE*Bs1*, *orf20* from Tn*916* in ICE-H1) was also removed to prevent the relaxase from nicking the origin of transfer (*oriT*) in the element and initiating rolling circle DNA replication with the element unable to excise from the chromosome. Rolling circle replication from *oriT* of ICE*Bs1* that is unable to excise causes a dramatic drop in host cell viability [44,45] and we wished to prevent this contribution to cell death. For simplicity, we refer to this hybrid element as ICE-H1-Δ*attR* (Rep-) (Materials and Methods), noting that although usually not indicated, it is also missing *orf20*, the gene that encodes the relaxase.

Similar to the effects of ICE-H1, activation of ICE-H1-Δ*attR* (Rep-) led to a decrease in cell growth (Fig 3A) and viability (Fig 3B and 3C). This decrease was apparent within one to two hours after activation of ICE-H1-Δ*attR* (by inducing expression of Pxyl-*rapI*) (Fig 3A, 3C). By three hours after induction, the relative viability of cells with ICE-H1-Δ*attR* (Rep-) was ~3000-fold lower than that of cells with the uninduced element (Fig 3B and 3C). The drop in viability caused by ICE-H1-Δ*attR* (Rep-) was ~30-fold more severe than that caused by ICE-H1 that was capable of excision and replication (Fig 2B). The less severe phenotype caused by ICE-H1 (capable of excision and replication) was likely due, at least in part, to the loss of the extrachromosomal form of this element following excision and then the selective advantage of cells that had lost the element.

Based on these results, we conclude that neither excision, nor nicking by the relaxase, nor rolling circle replication (initiated by the relaxase) are required for growth arrest or cell death. These phenotypes must be caused by other genes in ICE-H1.

### Multiple genes in Tn*916*, including *orf16* and *orf17*, contribute to the growth arrest and killing caused by activation of the ICE*Bs1*-Tn*916* hybrid ICE-H1

Using a series of deletions in ICE-H1-Δ*attR* (Rep-), which cannot excise from the chromosome or initiate autonomous replication, we found that several genes contributed to the growth arrest and cell death phenotypes caused by activation of the element. We monitored the growth of host strains containing ICE-H1-Δ*attR* (ELC1076), and ICE-H1-Δ*attR* (Rep-) with deletions of: Δ*orf23-22* (ELC1945), *Δorf21* (ELC1916), Δ*orf19* (ELC1915), Δ*ardA* (ELC1707), *Δorf17* (ELC1419), Δ*orf16* (ELC1420), Δ*orf17*-*16* (ELC1942), Δ*orf15* (ELC1418), Δ*orf14* (ELC1708), and Δ*orf13* (ELC1705). As above, each element could be activated by expression of Pxyl-*rapI*.

We found that loss of both *orf16* (*virB4*-like; homolog of *conE* in ICE*Bs1*) and *orf17* (*virB3*-like; homolog of *conD* in ICE*Bs1*) (*Δorf17-16*) almost completely suppressed the growth arrest, at least out to four hours after activation of the element, as determined by monitoring the OD of induced cultures (Fig 3A). Loss of *orf16* alone had a partial effect and loss of *orf17* alone had little or no effect (Fig 3A). These results could indicate that *orf16* and *orf17* are partly redundant for causing growth arrest. However, because the Orf17 homolog (ConD) from ICE*Bs1* affects the subcellular location of the Orf16 homolog (ConE) [46], we suspect that these two proteins work together.

No other deletions caused improved growth following element activation (Fig 3A). However, a few genes contributed to cell death, including *orf17* and *orf16*. Strains missing *orf17-16*, *orf21* (encoding the coupling protein *virD4*-like; *conQ* in ICE*Bs1*), *orf19* (essential for conjugation; same predicted topology as the *conC* gene product in ICE*Bs1*), or *orf15* (*virB6*-like; *conG* in ICE*Bs1*) all had an increase in viable cells three hours after activation, relative to that of the parent strain containing ICE-H1-Δ*attR* (Rep-) (Fig 3B). Notably, no single gene deletion fully restored viability of cells containing ICE-H1-Δ*attR* (Rep-), indicating that multiple Tn*916* genes contribute to death of host cells. We decided to focus on *orf17* and *orf16* due to their requirement for the growth arrest caused by activation of ICE-H1-Δ*attR* (Rep-) and their effect on cell viability.

In contrast to the almost complete restoration of cell growth in the *Δorf17-16* mutant, there was still a large drop in cell viability (Fig 3B). Deletion of both *orf16* and *orf17* caused a delay in the drop in viability as measured by CFUs, relative to that caused by ICE-H1-*ΔattR* (Rep-) (Fig 3C). One hour after activation of the element, the number of viable cells was ~100-fold greater in cells with ICE-H1-Δ*attR* (Rep-) Δ*orf17-16* compared to those with ICE-H1-Δ*attR* (Rep-) (Fig 3C). However, by three hours, the effect of Δ*orf17-16* was much less pronounced and the drop in CFUs was similar to that caused by ICE-H1-*ΔattR* (Rep-) (Fig 3B and 3C). The improvement in viability caused by Δ*orf17*-*16* was not due to polar effects on downstream genes. When Δ*orf17*-*16* was complemented with *orf17*-*16* at an ectopic site (*lacA*::P*xis orf17*-*orf16*, ELC1550), the growth defects were completely restored to the levels exhibited by ICE-H1-Δ*attR* (Rep-) (Fig 3B).

We hypothesized that the effect Orf16 was having (in combination with Orf17) on the host cell might be due to its activity as a VirB4-like ATPase. This was not the case. We monitored the impact of activation of an element containing a mutation affecting the predicted Walker A motif of Orf16 (K477E) that should eliminate ATPase activity. Although the *orf16*(*K477E*) mutation abolished conjugative transfer of Tn*916* and ICE-H1, the phenotypes caused by the point mutation with respect to cell growth and viability were indistinguishable from those caused by wild type *orf16* (Fig 3B).

Our results indicated that together, Orf16 and Orf17 are largely responsible for the arrest in cell growth, and partially responsible for the cell death caused by expression of the conjugation genes from Tn*916* that are present in ICE-H1. Based on these phenotypes, we decided to further analyze the effects of *orf16* and *orf17* on bacterial cells.

### *orf17* and *orf16* together are sufficient to cause growth arrest and a drop in viability

We found that expression of *orf16* and *orf17* together was sufficient to cause growth arrest in the absence of other Tn*916* genes. In strains devoid of any ICEs, we placed *orf17*, *orf16*, or *orf17*-*16* together under the regulatory control of P*xis* from ICE*Bs1* at an ectopic site, *lacA* (strains ELC1494, ELC1491, and ELC1496, respectively). A strain containing the vector with no genes inserted (ELC1495) was used as a control. These strains all contained the genes required for regulation of P*xis* (*immR*, *immA*, and Pxyl-*rapI*). We monitored effects of expression of *orf17* and-or *orf16* on cell growth and viability.

**Fig 4:**
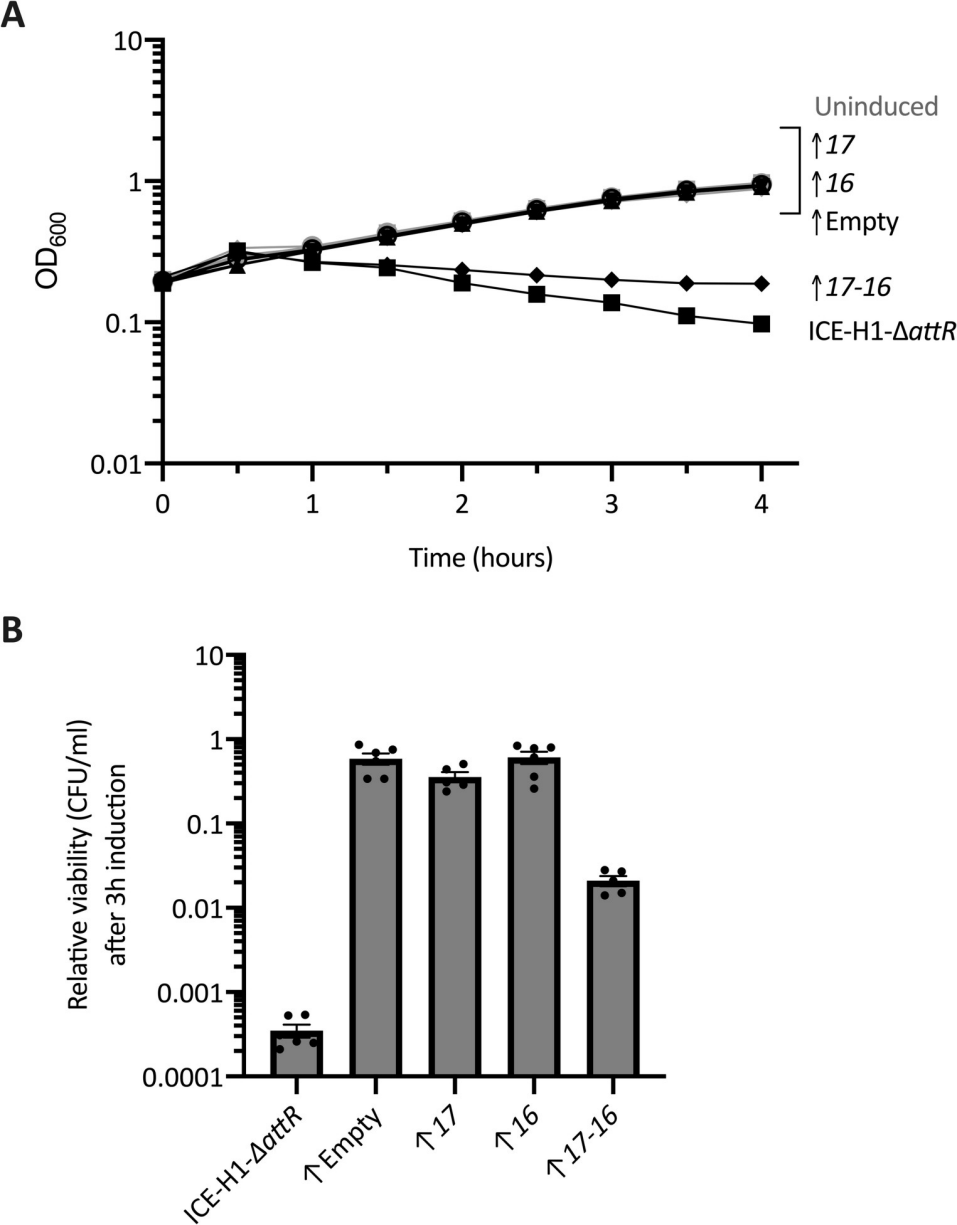
Orf17-16 are sufficient to cause arrest of cell growth. Strains containing overexpression alleles (indicated in the figure with an upwards arrow) of *orf17* (downward triangle, ELC1494), *orf16* (upward triangle, ELC1491), *orf17*-*16* (diamonds, ELC1496), or an empty vector (open circles, ELC1495) and a strain containing ICE-H1-Δ*attR* (Rep-) (squares, ELC1076) were grown in minimal medium with arabinose to early exponential phase. At an OD_600_ of ~0.2 (time = 0), cultures were split into inducing (+1% xylose to stimulate *rapI* expression) and non-inducing conditions. Data from three or more experiments are presented as averages (**A**) or individual data points (**B**), and error bars represent standard error of the mean. **A)** Growth was monitored by OD_600_ for three hours. Black lines indicate growth of the induced cultures; gray lines (difficult to see as they are clustered in the set of strains at the top of the graph) indicate growth of uninduced cultures. The growth curve of ELC1076 {containing ICE-H1-Δ*attR* (Rep-)} from Fig 3A is included as reference. Error bars could not always be depicted due to the size of each data point. **B)** The relative CFUs/ml of cultures after three hours of element induction was calculated as the number of CFUs formed by the induced culture, divided by that from the uninduced culture (a value of “1” indicates there is no change in CFUs with induction). Results from the overexpression of *orf17*-*orf16* were significantly different from the empty vector control based on P < 0.05 in unpaired two-tailed T-tests.

We found that by one hour after expression of *orf17* and *orf16* together, cell growth had decreased relative to that of no expression or expression of each gene separately (Fig 4A). The arrest in cell growth was similar to that caused by activation of ICE-H1-Δ*attR* (Rep-), although the decrease in OD was more severe following activation of ICE-H1-Δ*attR* (Rep-) (Fig 4A). In contrast, expression of *orf17* or *orf16* individually had little or no effect on cell growth (Fig 4A).

Expression of *orf17* and *orf16* together caused an approximately 50-fold drop in cell viability three hours after induction of expression (Fig 4B). This decrease in viability was less severe than that caused by ICE-H1-Δ*attR* (Rep-) (~2,000-fold, Fig 3B), indicating that *orf17* and *orf16* contribute to the drop in viability following induction of Tn*916* genes in ICE-H1, but that other Tn*916* genes are also required for the nearly 2000-fold drop in CFUs observed following expression of Tn*916* genes in ICE-H1-Δ*attR* (Rep-).

In contrast to the effects of *orf17* and *orf16* together, expression of each alone had relatively little effect on cell viability. There was an ~2–3 fold drop in cell viability after three hours of expression, but this occurred in the control that had no inserts (Fig 4B), indicating that this drop in viability likely resulted from the gene regulatory system (Pxyl-*rap*, *immR*, *immR*, and Pxis).

Together, our results indicate that expression of *orf17* and *orf16* together, in the absence of any other Tn*916* genes, is sufficient to cause growth arrest and cell death of *B*. *subtilis*. We suspect that *orf17* is needed for the proper expression of *orf16*. This is by analogy to the homologous genes *conD* (*orf17*) and *conE* (*orf16*) in ICE*Bs1* where ectopic expression of *conE* (*orf16*) is improved in the presence of the upstream gene *conD* (*orf17*), likely due to translational coupling [47]. Alternatively, or in addition, both proteins may be required to interact with one or more host components thereby leading to growth arrest and cell death. In the context of ICE*Bs1*, ConD assists in localizing ConE, a cytoplasmic protein, to the membrane [46]. We suspect that a similar interaction is occurring in the context of Tn*916*, although we do not know if this interaction is needed for the observed cellular phenotypes.

In contrast to the deleterious effects of Orf17 and Orf16 encoded by Tn*916* on cell growth and viability, the homologs encoded by ICE*Bs1*, ConD (19% identity, 35% similarity to Orf17) and ConE (23% identity, 43% similarity to Orf16) do not cause similar phenotypes. From this and previous works, we know that activation of ICE*Bs1* does not cause growth arrest and death (Fig 3). Furthermore, previously published ectopic expression constructs of ConD and ConE did not cause such effects [47]. Although these homologs almost certainly perform similar functions during conjugative transfer of the two elements, the differences in the sequences enable the Tn*916* products to have dramatic effects on host cell physiology. We do not know which parts of the sequence of either protein from Tn*916* contribute to growth arrest or cell death.

### Host-encoded *yqaR* is necessary for *orf17-16*-caused growth arrest and cell death

We set out to identify host genes that are required for the cell death caused by expression of *orf16* and *orf17* from Tn*916*. Because expression of *orf17*-*16* causes cell death, we simply isolated suppressor mutations that enable cell survival. We expected to get mutations that prevent expression of functional *orf17*-*16* from P*xis*. These could include mutations in *orf17*-*16* themselves, or in the regulatory genes (*rapI* and *immA*) needed for inactivation of the repressor ImmR and derepression of P*xis*. To reduce the frequency of mutations in these genes, we enriched for survivors in a strain that contained two copies each of *orf17*-*16*, *rapI*, and *immA* (ELC1760). In addition, we included a P*xis*-*lacZ* fusion that would be derepressed similarly to P*xis*-*orf17*-*16*. In this way, we could monitor production of ß-galactosidase to eliminate mutants in which P*xis* could not be expressed.

To ensure that we isolated independent mutants, we grew eighteen separate cultures of ELC1760 and isolated one candidate from each culture. Cells were grown in defined minimal medium (with 1% arabinose) and expression of P*xis*-*orf17*-*16* was induced with 1% xylose and grown overnight (approximately 18 hours). Cultures were diluted and this process was repeated 1–2 times to enrich for suppressor mutants (Materials and Methods). Cells were then plated onto LB agar plates under non-inducing conditions and candidate mutants were colony-purified and checked for presence of all antibiotic resistance markers. Additionally, we confirmed these isolates properly activated P*xis*-*lacZ* when streaked on LB plates containing X-gal (5-bromo-4-chloro-3-indolyl-β-D-galactopyranoside) and 1% xylose, indicating that the RapI-driven induction of P*xis* was functional (and likely *orf17* and *orf16* were still being expressed). We isolated 18 independent suppressor mutants, one from each of the separate cultures. Genomic DNA from each of these 18 mutants was used for whole genome sequencing to locate chromosomal mutations.

DNA sequencing indicated that 15 of the 18 mutants were cured of *skin* (*sigK* intervening), a genetic element that interrupts *sigK*. *sigK* encodes the mother-cell specific sigma factor (σ^K^) that is required for sporulation [48,49]. The remaining three mutants each contained a frameshift mutation {either (A)_8→7_ at nucleotide 50 of 465, or (T)_7→6_ at nucleotide 450} in *yqaR*, a gene in *skin*. *Skin* is a remnant of a prophage [50] and contains several homologs of genes in PBSX, a co-resident defective prophage in *B*. *subtilis* [51]. Although *yqaR* is encoded between homologs of a PBSX transcription factor (*yqaQ*) and phage terminase proteins (*yqaST*), there are no homologs of *yqaR* in PBSX [51]. Little has been reported about YqaR, although it was identified as a membrane protein found in *B*. *subtilis* spores [52]. It is expressed in a variety of growth conditions [53], indicating that normal expression of this gene does not cause growth arrest or cell death.

**Fig 5:**
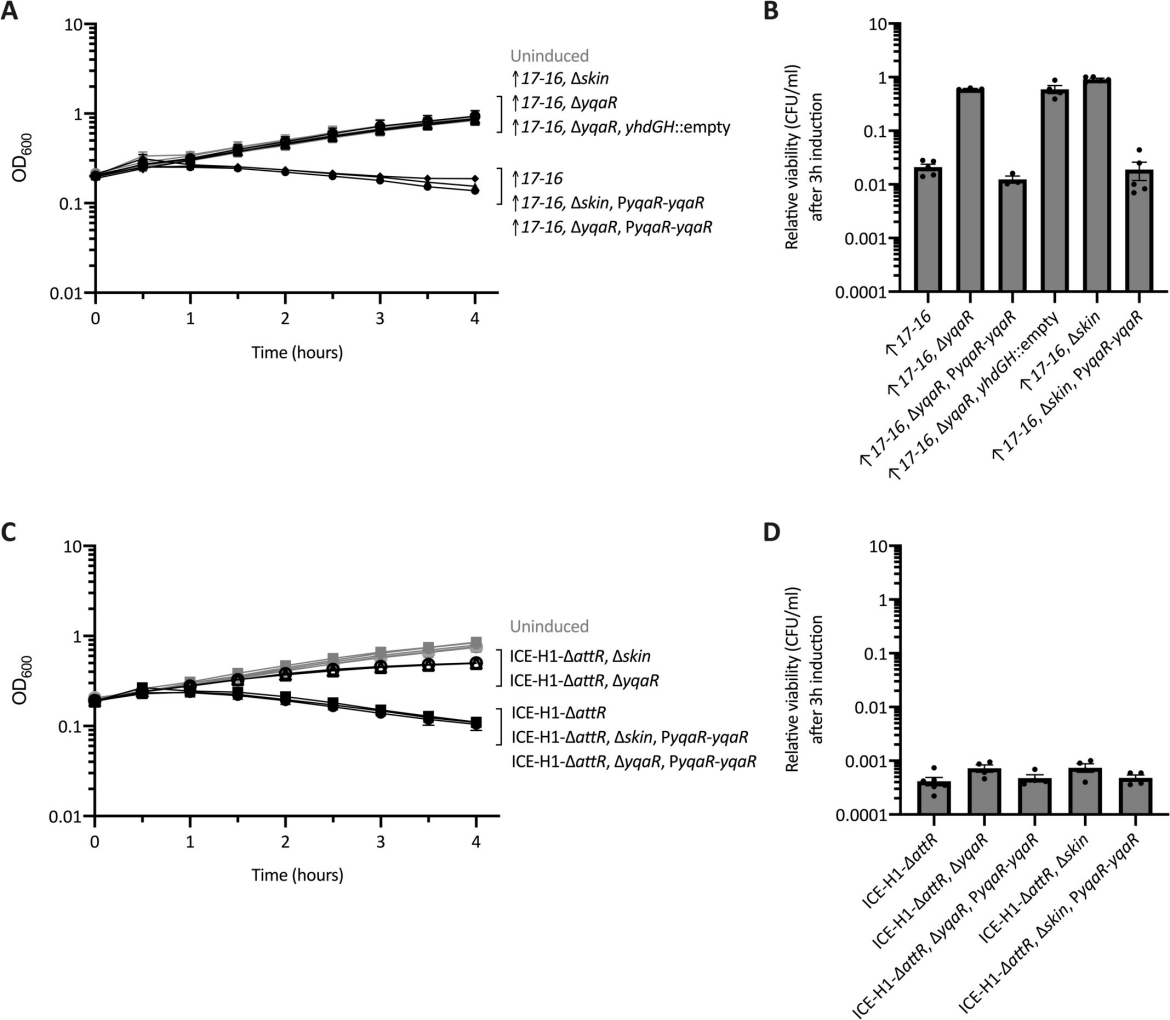
Effects of *skin*-encoded *yqaR* on growth arrest caused by *orf17-16* and ICE-H1. Indicated strains were grown in defined minimal arabinose medium to early exponential phase. At time = 0 hours, when cultures were at an OD_600_ ~0.2, cultures were split into inducing (+1% xylose to stimulate *rapI* expression and de-repression of *orf17-16* or of the indicated ICE hybrid) and non-inducing (no xylose) conditions. Data from three or more experiments are presented as averages (**A, C**) or individual data points (**B, D**), and error bars represent standard error of the mean. **A,B)** Strains contained *orf17*-*16* overexpression alleles with the following additional alleles: WT (diamonds, ELC1496), Δ*skin* (open circles, ELC1891), Δ*yqaR* (open upward triangles, ELC1892), Δ*yqaR yhdGH*::empty (open downward triangles, ELC1918), Δ*skin* P*yqaR*-*yqaR* (closed circles ELC1903), and Δ*yqaR* P*yqaR*-*yqaR* (closed upward triangles, ELC1904). **C,D)** Strains contained ICE-H1-Δ*attR* with the following additional alleles: WT (squares, ELC1076), Δ*skin* (open circles, ELC1908), Δ*yqaR* (open upward triangles, ELC1856), Δ*skin* P*yqaR*-*yqaR* (closed circles, ELC1909), and Δ*yqaR* P*yqaR*-*yqaR* (closed upward triangles, ELC1911). **A,C)** Growth was monitored by OD_600_ for three hours. Black lines indicate growth of the induced cultures; gray lines (some are difficult to see as they are clustered in the set of strains at the top of the graph) indicate growth of uninduced cultures. Error bars could not always be depicted due to the size of each data point. **B,D)** The relative CFUs/ml of cultures after three hours of induction of *orf17-16* or the indicated element was calculated as the number of CFUs formed by the induced culture, divided by that from the uninduced culture (a value of “1” indicates there is no change in CFUs with induction). In panel B: Significant differences based on P < 0.05 in unpaired two-tailed T-tests include the overexpression of *orf17*-*orf16* compared to: Δ*skin*, Δ*yqaR*, and Δ*yqaR yhdGH*::empty. The small differences apparent in panel D are not statistically significant.

We reconstructed strains to verify that loss of *yqaR* or *skin* suppressed the phenotypes caused by overexpression of *orf17-16*. These strains contained the xylose-inducible *orf17*-*16* (Pxyl-*orf17-16*) and a deletion of either *skin* (ELC1891) or *yqaR* (Δ*yqaR*::*cat*) (ELC1892). Growth and viability were monitored before and after expression of *orf17*-*16*, essentially as described above. The growth and viability of these strains under inducing conditions was indistinguishable from non-inducing conditions (Fig 5A and 5B), indicating that deletion of *yqaR* or *skin* suppressed growth arrest and cell killing caused by expression of *orf17*-*16*. In other words, *yqaR* of the *skin* element was needed for *orf17-16*-mediated growth arrest and cell death.

We found that *yqaR* was the only gene in *skin* needed for the killing caused by expression of *orf17*-*16*. Introduction of a copy of *yqaR*, expressed from its predicted promoter, P*yqaR*, at an ectopic site (*yhdGH*) in the absence of *skin* completely restored the growth defect and cell death caused by expression of *orf17*-*16* (Fig 5A and 5B). This construct also complemented the *ΔyqaR* mutation (Fig 5A and 5B). Together, these results indicate that *yqaR* is necessary for effects of *orf17*-*16* on cell growth and viability, and that of all the genes in *skin*, it is sufficient for these effects.

### Deleting *yqaR* partially relieves growth defects caused by activation of ICE-H1

We wished to determine if *yqaR* (or *skin*) contributed to the growth arrest and cell death caused by ICE-H1. If the other Tn*916* genes present in ICE-H1 functioned similarly to *orf17-16* in affecting cell viability, then loss of *yqaR* should similarly suppress those effects. However, if *yqaR* function was limited to *orf17-16*, then loss of *yqaR* would only partly suppress the cell death caused by activation of ICE-H1.

We found that deletion of *yqaR* (or *skin*) in strains containing the excision- and replication-deficient ICE-H1-Δ*attR* (Rep-) partially, but not fully, relieved the growth and viability defects caused by activation of the hybrid ICE. We monitored the growth and viability of strains containing ICE-H1-Δ*attR* (Rep-) with either Δ*skin* or Δ*yqaR*::*cat* (ELC1908 and ELC1856, respectively) following element activation. Cell growth was largely restored, although there was a consistent and small decrease in OD relative to that of the uninduced cultures (Fig 5C). The introduction of P*yqaR*-*yqaR* into both Δ*skin* or Δ*yqaR*::*cat* strains (ELC1911 and ELC1909, respectively) was sufficient to restore the growth defects indicating that the suppressive phenotype was caused by loss of *yqaR* and that *yqaR* is the only *skin*-encoded gene necessary for growth arrest and the decrease in optical density.

In contrast to the effect on cell growth, loss of *yqaR* or *skin* only had a minor effect on cell viability following activation of ICE-H1-Δ*attR* (Rep-) (Fig 5D). The *ΔyqaR* or Δ*skin* mutants consistently had slightly more colonies (~2-fold) post-induction than the wild type host strain. The strains in which P*yqaR*-*yqaR* was introduced to complement the deletions behaved indistinguishably from the wild type host. These results indicate that *yqaR* does not greatly contribute to the killing effects caused by other Tn*916* genes in the context of ICE-H1-Δ*attR* (Rep-), despite its involvement in growth arrest and killing mediated by *orf17-16*.

### Construction of a fluorescent reporter system to visualize Tn*916*-activated cells

These heterologous expression systems revealed that activation of Tn*916* genes negatively impacts population growth and cell viability. However, these are artificial systems in which Tn*916* genes in ICE-H1 are regulated quite differently from that in their normal context in Tn*916*. Therefore, we wished to evaluate the effects of normal, endogenous activation of Tn*916* on host cells. Because Tn*916* is only activated in a small fraction of cells, we could not do these analyses on a population level. Instead, we analyzed behavior of single cells in which Tn*916* had been activated.

**Fig 6:**
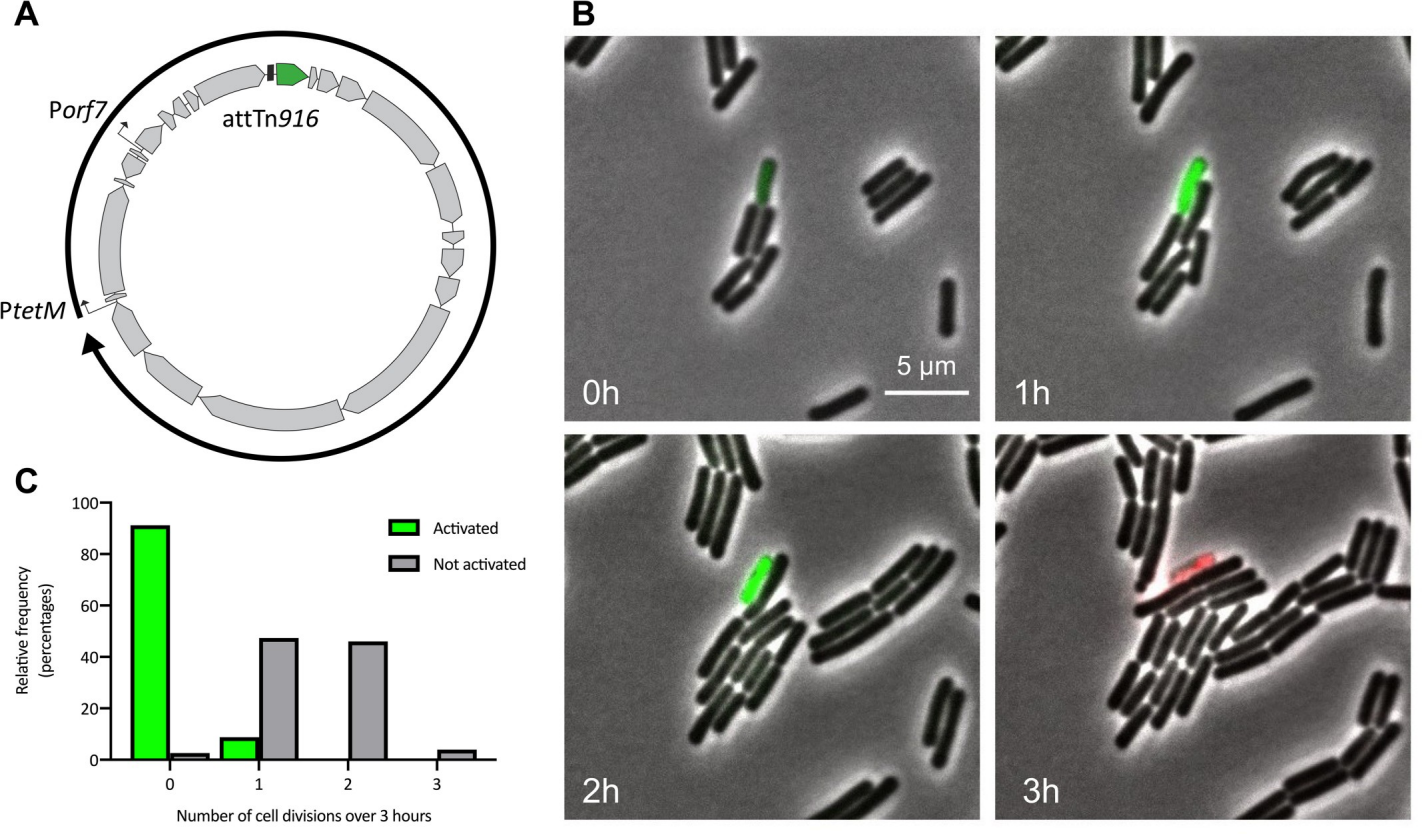
Tn*916*-activated *B*. *subtilis* cells exhibit growth defects. **A)** The fluorescent reporter system for monitoring Tn*916* excision (Tn*916*-*gfp*). A circular genetic map of Tn*916* is shown, with gray boxes with arrows indicating Tn*916* open reading frames, bent arrows representing promoters, and a black box representing the circular attachment site, *att*Tn*916*. P*tetM* and P*orf7* are predicted to drive expression of *orf24*-*orf13* following excision and circularization of the element (reviewed in [4]). *gfpmut2* (shown in green) was inserted upstream of *orf24* such that it is expressed following element excision. **B,C)** Cells containing Tn*916*-*gfp* integrated between *yufK*-*yufL* (ELC1458) were grown in minimal glucose medium to late exponential phase with 2.5 μg/ml tetracycline to stimulate Tn*916* excision. At time = 0 h, cells were spotted on minimal glucose agarose pads containing 2.5 μg/ml tetracycline, 0.1 μM propidium iodide, and 0.035 μg/ml DAPI. Cells were monitored by phase contrast and fluorescence microscopy for three hours. **B)** A representative set of images from these experiments. GFP (green) was produced in cells in which Tn*916* was activated and excised from the chromosome. Propidium iodide (red) indicates dead cells. **C)** Histogram displaying the relative frequency (percentage) of Tn*916*-*gfp* activated cells that underwent the indicated number of cell divisions, compared to non-activated (GFP-negative) cells. DAPI and phase contrast were used for monitoring cell division events.

To identify individual cells in which Tn*916* had been activated and to monitor potential effects of Tn*916* in its native context on cell growth and viability, we generated a fluorescent reporter to track element activation in single cells. We took advantage of the fact that the DNA processing and conjugation genes in Tn*916* are not expressed until the element excises from the chromosome and forms a circular intermediate [33]. Circularization allows promoters on the “right” side of the element to be joined with those genes encoded on the “left” side of the element (as drawn in Fig 1A). By inserting *gfpmut2* upstream of *orf24* in Tn*916* (Tn*916*-*gfp*), we generated a reporter system in which cells only fluoresce green when the element has been activated and excised (Fig 6A).

Insertion of *gfp* near the left end of Tn*916*-*gfp* did not abolish excision. We found that after 3 hours of growth in the presence of tetracycline (to stimulate excision), Tn*916-gfp* had excised in ~0.1% of cells, as measured by qPCR and by counting cells that produced GFP. These results indicated that this reporter is a reliable method to monitor Tn*916* activation in single cells.

### Growth defects caused by Tn*916* activation in *B*. *subtilis* are observable on the single-cell level

By using this fluorescent reporter system, we found that *B*. *subtilis* cells in which Tn*916* was activated under its endogenous regulatory system underwent limited to no cell divisions and frequently lysed. Cells containing Tn*916*-*gfp* (ELC1458) were grown in a minimal medium to early exponential phase, then treated with tetracycline to stimulate activation. Three hours later, we visualized cells microscopically, tracking cells that had activated Tn*916*-*gfp* (green, Tn*916*-*gfp* on) and comparing them to cells that had not activated Tn*916*-*gfp* using time-lapse microscopy over the course of three hours.

We tracked 34 cells in which Tn*916*-*gfp* had excised (GFP on) (Fig 6B, S1 and S2 Videos). Of these 34 cells, 31 (91%) did not undergo any further cell divisions and 3 (9%) divided once (Fig 6C). For comparison, we tracked 76 cells in which Tn*916*-*gfp* had not excised (GFP off). Of these 76 cells, only 2 (3%) did not divide and 74 (97%) underwent one or more cell divisions.

We also found that cells containing an activated Tn*916* often lysed (S1 and S2 Videos). We used propidium iodide (PI) to monitor the viability of these cells [54]. Propidium iodide only penetrates cells with damaged membranes and is widely used as an indicator of cell death. Of the 34 cells that had activated Tn*916*-*gfp*, 91% stained with PI during the course of the experiment (Table 1). In contrast, only 3% of the 76 cells that had not activated Tn*916*-*gfp* were PI-positive or had a PI-positive daughter cell by the end of the time lapse. The division frequencies and cell lysis were similar for cells in which Tn*916* was already activated (GFP on) prior to placement on the agarose pads compared to those cells in which Tn*916* appeared to become activated (GFP-off to GFP-on) while on the agarose pad. Results from either case are combined in Fig 6C and Table 1.

**Table 1:** Growth defects caused by an active Tn*916* in various host strains^a^.

*B*. *subtilis* Tn*916*-*gfp* integration site^b^	Tn*916* activation state; mutations	# cells	Cells that divided	PI-stained cells
*yufK*-*yufL*	Active	34	9%	91%
	Non-active	76	97%	3%
	Active; no tetracycline	20	5%	100%
	Active; Δ*orf17*-*16*	36	3%	83%
	Active; Δ*yqaR*	23	13%	65%
*ykuC*-*ykuB*	Active	20	< 5%	95%
*nupQ*-*maeN*	Active	28	7%	79%

^a^Strains were grown in defined minimal glucose medium to late exponential phase with 2.5 μg/ml tetracycline to stimulate Tn*916* excision (unless otherwise indicated). At time = 0 h, cells were spotted on minimal glucose agarose pads containing 2.5 μg/ml tetracycline, 0.1 μM propidium iodide, and 0.035 μg/ml DAPI. Cells were monitored by phase contrast and fluorescence microscopy for three hours. Cells that contained an active (GFP-positive) or non-active (GFP-negative) Tn*916*-*gfp* cells were monitored and the number of cell divisions was counted. Cells were counted as dead if either they or any daughter cells turned PI-positive during the 3-hour time lapse.

^b^Host strains examined: Tn*916*-*gfp* was located in 3 different chromosomal locations: between *yufK*-*yufL* (ELC1458), *ykuC*-*ykyB* (LKM20), and *nupQ*-*maeN* (LKM18), and two mutant strains with Tn*916*-*gfp* integrated between *yufK*-*yufL* with the indicated deletions: Δ*orf17*-*16* (ELC1512) and Δ*yqaR* (ELC1857).

We were concerned that the growth defect and loss of viability that we observed might be due to treatment with tetracycline. This was not the case. Neither growth arrest nor cell death were dependent on the presence of tetracycline. Tetracycline modestly enhances, but is not necessary for Tn*916* activation [32,33,35–37]. To confirm that the presence of the antibiotic was not impacting the growth of activated cells, we monitored the growth and viability of Tn*916* activated cells without the addition of tetracycline in conditions otherwise identical to those described above. We identified 20 cells that had activated Tn*916*-*gfp* (in the absence of tetracycline). Of these 20 cells, 19 (95%) did not divide, and all 20 (100%) lysed during the course of the experiment (Table 1). These results are indistinguishable from those observed with tetracycline added. Because tetracycline modestly enhances Tn*916* activation, consequently increasing the number of activated cells to track, it was included for experiments described below.

We found that the growth and viability defects caused by excision of Tn*916*-*gfp* from the insertion site between *yufK* and *yufL* were not unique to the particular Tn*916* genomic insertion site. Tn*916* can insert into many sites in AT-rich regions in the bacterial chromosome [40,55–57]. Therefore, we monitored the growth of cells in which Tn*916*-*gfp* was located at two different locations on the chromosome, one inserted between *ykuC* and *ykyB* (LKM20) and one between *nupQ* and *maeN* (LKM18). We observed similar results of limited divisions and frequent lysis for these two insertion sites compared to the insertion between *yufK* and *yufL* (Table 1), indicating these results were not dependent on the integration site of Tn*916*.

Together, these results indicate that cells in which Tn*916* became activated were unable to divide and lost viability. These results are consistent with those we observed on the population-level using the ICE*Bs1*-Tn*916* hybrid (ICE-H1), that activates Tn*916* genes in the majority of cells in a population.

### Host cells lacking *orf17*-*16* or *yqaR* have growth defects following element activation

Because *yqaR* had relatively little effect on viability of cells with an activated ICE-H1 (Fig 5D), we anticipated that it would have little effect on cell death caused by Tn*916*. Similarly, we were interested in the effects of *orf17-16* in the context of Tn*916*. Under conditions identical to those described above, we monitored the number of divisions and viability of Δ*orf17*-*16* and Δ*yqaR* Tn*916-gfp* host cells (ELC1512, ELC1857, respectively). Loss of *orf17-16* or *yqaR* did not drastically increase the number of cell divisions of the activated host cells (Table 1), nor was there a dramatic change in cell viability (PI staining). Overall, these results indicated that these mutations did not enable restored growth or viability of activated cells, although the data from the microscopy assays lack the resolution necessary to detect small changes. These results further support the conclusion that Tn*916* has multiple mechanisms to manipulate host cell growth and viability beyond the relationship between Orf17-16 and YqaR.

### Deleting *yqaR* in Tn*916* host cells increases conjugation efficiency

The phenotypic interactions between *orf17-16* and *yqaR* indicated that *yqaR* (and *skin*) might have some effect on the function of Tn*916*. For example, *yqaR* might limit the ability of Tn*916* to efficiently move from cell-to-cell. Alternatively, Tn*916* might be manipulating the host through *yqaR*, perhaps enabling the formation of "mating bodies" [9], or the release of DNA [58] that would better enable spread of Tn*916*.

**Fig 7:**
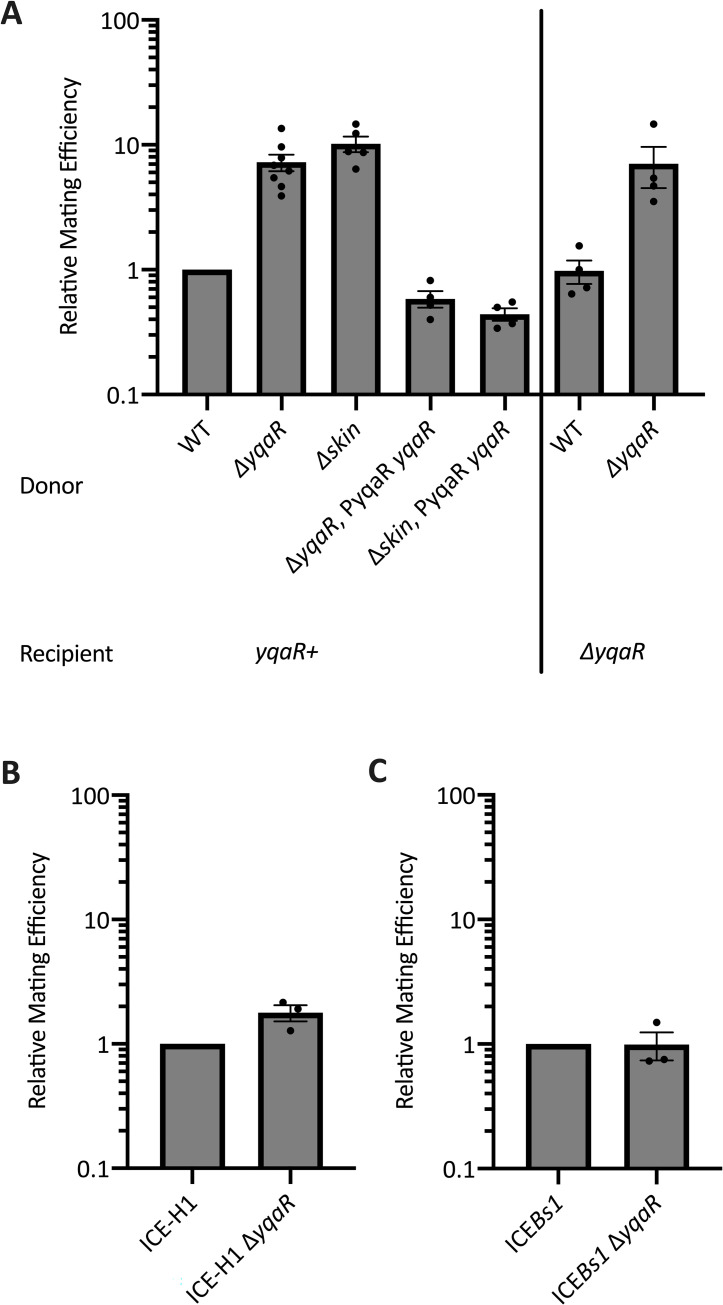
Effects of *yqaR* and *skin* on mating efficiency of Tn*916*. The indicated strains were grown to early exponential phase in LB medium. Activation of Tn*916* (**A**) was stimulated by adding 2.5 μg/ml tetracycline for one hour prior to mixing with the indicated recipients. ICE*Bs1* and ICE-H1 (**B, C**) were activated by addition of 1 mM IPTG for one hour prior to mixing with recipients. Data are shown from three or more independent experiments. Error bars represent standard error of the mean. Typical conjugation efficiencies in these experiments were: Tn*916* ~0.0005%, ICE-H1 ~1%, ICE*Bs1* ~1.5%. **A)** Tn*916* donors: WT (CMJ253), Δ*yqaR* (ELC1851), Δ*skin* (ELC1846), Δ*yqaR* P*yqaR*-*yqaR* (ELC1922), Δ*skin* P*yqaR*-*yqaR* (ELC1923) were mixed recipients: *yqaR*+ (ELC301) or Δ*yqaR* (ELC1854). Conjugation efficiencies (the number of transconjugants produced divided by the number of donors applied to mating) were normalized to those calculated for WT Tn*916* donors mated into *yqaR+* recipients, which were completed in parallel for each experimental replicate. The mating efficiencies of Tn*916* from wild-type, Δ*yqaR* PyqaR-*yqaR*, and Δ*skin* PyqaR-*yqaR* donors were significantly different than those from Δ*yqaR* and Δ*skin* donors based on P < 0.05 in ratio paired two-tailed T-tests. **B,C)** Indicated donors were mixed with *yqaR*+ (ELC301) recipients. (**B**) donors were ICE-H1 (ELC1213) or ICE-H1 Δ*yqaR* (ELC1843). (**C**) donors were ICE*Bs1* (JMA168), or ICE*Bs1* Δ*yqaR* (ELC1844). Conjugation efficiencies of Δ*yqaR* donors were normalized to that of the *yqaR*+ donor in experiments conducted in parallel. The difference in mating efficiency of ICE-H1 (panel B) from the Δ*yqaR* mutant compared to wild type was consistently about 2-fold, although this was not statically significant (P = 0.068) based on comparison in a ratio-pair two-tailed T-test. There was no significant difference comparing data from the two strains in panel C.

We found that *yqaR* limits transfer of Tn*916*. The mating efficiency of Tn*916* from donor cells with a null mutation in *yqaR* (ELC1851) was approximately 10-fold greater than that of their *yqaR*+ (CMJ253) counterparts (Fig 7A). However, deleting *yqaR* in recipient cells (ELC1854) did not cause detectable differences in mating efficiencies compared to *yqaR*+ recipients (ELC301), indicating that the effects of *yqaR* are specific to donors. In parallel experiments, we confirmed that no other genes in *skin* affected the mating efficiency of Tn*916*. Δ*skin* donor cells (ELC1846) had an increase in mating efficiency similar to that of Δ*yqaR* donor cells (Fig 7A). Additionally, complementing *yqaR* under its native promoter at an ectopic locus (*yhdGH*) restored Tn*916* mating efficiencies to WT levels (Fig 7A). These results indicate that the presence of *yqaR* negatively affects the transfer efficiency of Tn*916*.

We found that the effects of *yqaR* on Tn*916* conjugation are downstream of the activation step of the element’s lifecycle. Deleting *yqaR* did not alter the activation frequency of Tn*916*. Tn*916* had excised (and was therefore activated) in 0.79 ± 0.02% of Δ*yqaR* donors compared to 0.83 ± 0.02% of WT Tn*916* donors immediately prior to the start of the mating.

Deleting *yqaR* had less of an impact on the transfer efficiency of the hybrid conjugative element ICE-H1 than on that of Tn*916*. ICE-H1 Δ*yqaR* donors (ELC1843) mated only ~2-fold more efficiently than *yqaR*+ donors (ELC1213) (Fig 7B). We suspect that the increased activation frequency of ICE-H1 relative to that of Tn*916*, and consequently increased transfer efficiencies (and perhaps increased transcription of element genes) masks possible effects of *yqaR* on mating efficiencies.

Because ICE*Bs1* activation does not cause growth defects like those elicited by Tn*916*, we did not expect *yqaR* to influence ICE*Bs1* mating efficiencies. Indeed, Δ*yqaR* ICE*Bs1* donors (ELC1844) did not exhibit altered mating efficiencies compared to *yqaR*+ ICE*Bs1* donors (MMB766) (Fig 7C). This result confirms that *yqaR* is specifically interacting with Tn*916*-encoded genes, and not broadly impacting the transfer of conjugative elements.

Together, these results indicate that the YqaR-dependent growth defects caused by Tn*916* genes are not beneficial for efficient element transfer. Instead, it appears that *B*. *subtilis* encodes a mechanism to limit the spread of Tn*916* through a population of cells. However, these results do not elucidate any effects that other Tn*916*-mediated growth defects may have on transfer efficiency.

### Tn*916* activation causes growth defects in *Enterococcus faecalis*

Whereas *B*. *subtilis* is a convenient host for analyzing Tn*916* (e.g., [27–32]), it is not a natural host. Furthermore, homologs of YqaR are not found in any of its natural hosts (*Enterococcus*, *Streptococcus*, *Staphylococcus*, and *Clostridium* species). Therefore, we wondered if this killing elicited by activated Tn*916* is specific to *B*. *subtilis* or might also occur in a natural host.

**Fig 8:**
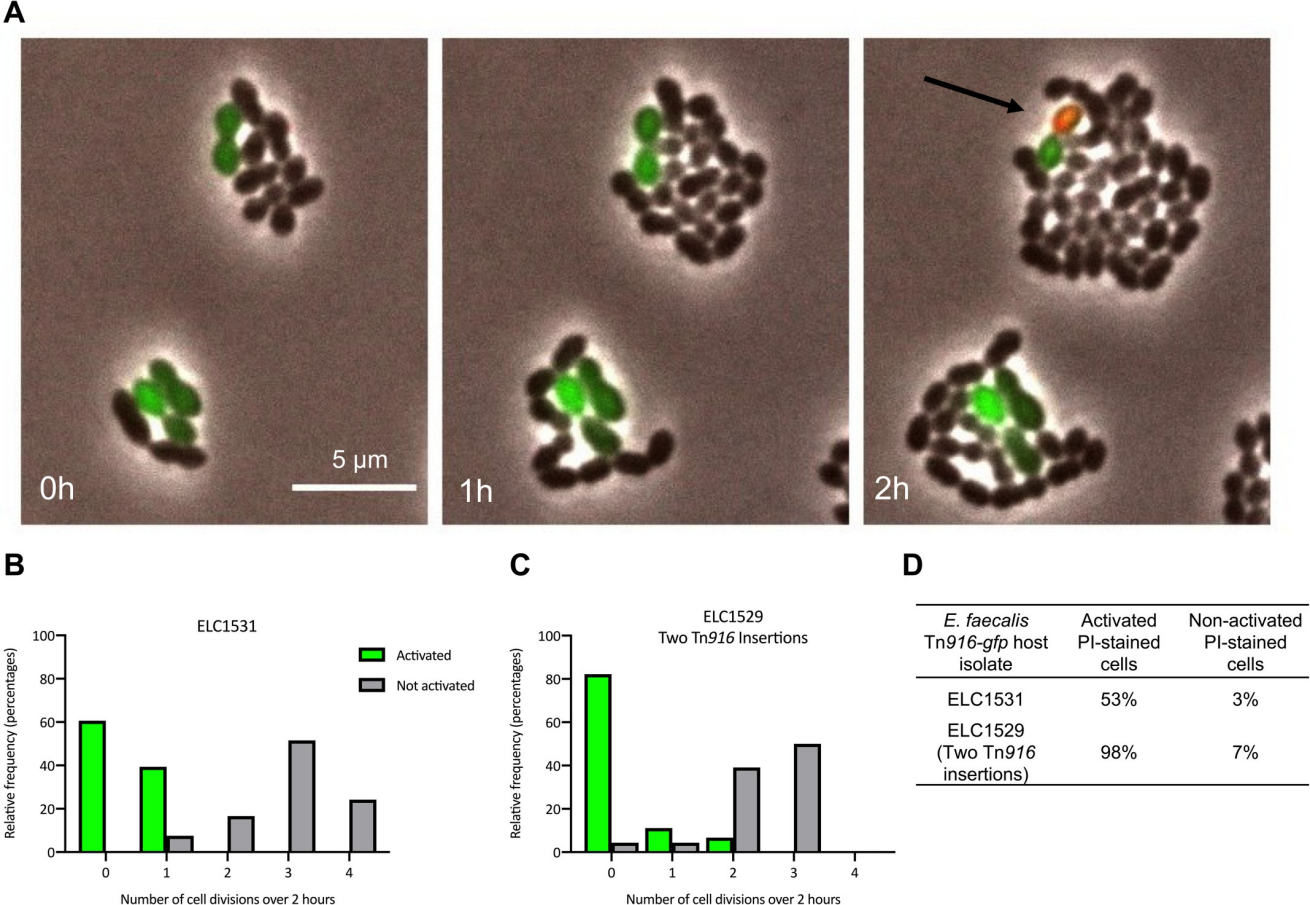
Activation of Tn*916* causes growth arrest and cell death in *E*. *faecalis*. Two separate isolates of *E*. *faecalis* containing Tn*916*-*gfp* were used to monitor effects of Tn*916* activation. *E*. *faecalis* strains ELC1531 and ELC1529 have one and two copies of Tn*916-gfp*, respectively (see Materials and Methods). Cells were grown in a rich M9 medium (Methods) to late exponential phase with 2.5 μg/ml tetracycline to stimulate Tn*916* excision. At time = 0 h, cells were spotted on M9 medium agarose pads containing 2.5 μg/ml tetracycline, 0.1 μM propidium iodide, and 0.5 μg/ml DAPI. Cells were monitored by phase contrast and fluorescence microscopy for two hours. **A)** A representative set of images monitoring ELC1531 cells with an activated copy of Tn*916*-*gfp* (GFP-positive). Similar results were observed with ELC1529. The black arrow in the final frame indicates a PI-stained, GFP-positive cell (appears reddish-yellow). **B,C)** Histograms displaying the relative frequency (percentage) of Tn*916*-*gfp* activated (GFP-positive) cells that underwent the indicated number of cell divisions, compared to non-activated (GFP-negative) cells for each isolate. Sixty-six activated (GFP-positive) and 66 non-activated (GFP-negative) cells for ELC1531 and 45 of each for ELC1529 were monitored. **D)** The frequency of cells that stained with PI (indicating cell death) or had a daughter cell become PI-positive was determined for both activated and non-activated cells during the 2-hour time lapse.

We found that Tn*916* activation-related growth defects and death also occur in *Enterococcus faecalis*, a natural Tn*916* host. We monitored the effects of Tn*916* activation in *Enterococcus faecalis*, the first-discovered host species of Tn*916* [19,20] using the same fluorescent reporter system described above. Tn*916*-*gfp* was mated into *E*. *faecalis* (ATCC 19433) and two independent transconjugants (strains ELC1531 and ELC1529) were isolated to evaluate the effects of activation (excision) of Tn*916*-*gfp* from different chromosomal integration sites (Materials and Methods). ELC1531 had a single copy and ELC1529 had two copies of Tn*916-gfp* integrated in the chromosome (see below). *E*. *faecalis* does not grow on the defined minimal medium used for *B*. *subtilis* microscopy; instead, we used a rich medium to grow and visualize *E*. *faecalis* during live cell imaging. Each *E*. *faecalis* strain containing Tn*916-gfp* (strains ELC1531 and ELC1529) was grown to early exponential phase (Materials and Methods), tetracycline (2.5 μg/ml) was added to stimulate excision of the element, and after an hour of growth with tetracycline, cells were visualized for two hours using time-lapse microscopy (Fig 8A and S3 Video).

Cells in which Tn*916* had excised (green) had a decrease in cell divisions and were stained with PI more frequently than neighboring non-activated cells (Fig 8, S3 Video). We tracked 66 cells that were producing GFP (had an excised Tn*916-gfp*; in strain ELC1531,): forty cells (61%) underwent no divisions, 26 cells (39%) underwent one division. Thirty-five cells (53%) appeared to lose viability based on staining with PI during the time lapse. In contrast, of the 66 non-activated cells that were tracked, 100% underwent one or more divisions and only 2 (3%) became PI-positive or had progeny that became PI-positive.

We tracked 45 activated cells from the other *E*. *faecalis* isolate (strain ELC1529): 8 (18%) underwent one or more divisions and 98% appeared to lose viability based on staining with PI during the time lapse. Of the 45 non-activated cells that were tracked, 43 (96%) underwent one or more divisions and only 3 (7%) became PI-positive.

The growth defects were most pronounced in ELC1529, which has two copies of Tn*916* in the chromosome **(**Materials and Methods**)**. We suspect that having two copies of Tn*916* exacerbated the growth phenotypes following Tn*916* activation. It is likely that the activation and excision of one copy of Tn*916* leads to activation and excision of the second copy [36,59,60]. Thus, having two copies of Tn*916* in a host cell likely leads to higher expression levels of detrimental gene(s), which could be deleterious for the host. This result is interesting given that Tn*916* does not possess any known mechanisms to prevent acquisition of multiple copies in a single host, such as exclusion systems that are used by conjugative plasmids and some ICEs [10,61,62].

Although we did not identify a homolog of *yqaR* in *E*. *faecalis*, we confirmed that the deleterious interactions between Tn*916* and *E*. *faecalis* host cells were independent of *orf17*-*orf16*. We mated a Tn*916-gfp* (*orf17*-*orf16*) mutant from a *B*. *subtilis* donor into *E*. *faecalis* (ELC1696; the *orf17-16* were provided ectopically in the donor; Materials and Methods). We monitored the growth of 23 activated cells. Twelve (52%) underwent one or more division and 11 (48%) appeared to lose viability based on PI staining. These results are similar to those observed for ELC1531, indicating that deleting *orf17*-*orf16* from Tn*916-gfp* did not greatly improve growth or viability of host cells following activation. It is possible that these experiments do not possess the resolution necessary to detect growth improvements, as noted for *B*. *subtilis* microscopy experiments described above. However, these results highlight that Tn*916* possesses mechanisms independent of *orf17*-*orf16* to modulate growth and viability of multiple host species. We suspect that at least some of these mechanisms function similarly in different hosts.

## Discussion

The experiments described here demonstrate that activation of Tn*916* causes growth arrest and cell death, both in *B*. *subtilis* and a natural host *E*. *faecalis*. We suspect that these effects were previously undetected due to the low activation frequency of the element. We were able to detect the growth arrest and cell death caused by Tn*916* by studying a hybrid ICE that can be activated in a large fraction of cells in a population, and by analyzing the low activation frequency Tn*916* in single cells using a fluorescent reporter.

Two previous reports contained results indicating that when activated, Tn*916* was deleterious to the host cell. We previously noted that the percentage of cells in a population that contained a circular (excised) Tn*916* decreased over time, leading us to speculate that excision of Tn*916* might cause some deleterious effect on cell growth [32]. Additionally, a previous report found that *E*. *faecalis* host cells containing a Tn*916* mutant with increased excision frequencies (due to mutations in the regulatory region upstream of *tetM*) had decreased fitness relative to cells containing wild type Tn*916* [63]. The authors hypothesized that the decreased fitness was due to increased production of TetM, possibly slowing protein production, and an additional cost of the hyper-conjugative phenotype of the mutant [63]. We suspect that in both these studies [32,63], the decreased fitness was due to the growth arrest and cell killing described here.

### Interactions between a conjugative element (Tn*916*) and a defective prophage-like element (*skin*)

We found that multiple Tn*916*-encoded genes contribute to the growth arrest and cell death. Growth arrest and cell death caused by Orf17-16 was dependent on *yqaR*, a gene found within the defective phage-like element *skin*. Loss of *yqaR* (or *skin*) leads to an increase in Tn*916* conjugation, indicating that one function of *yqaR* might be to limit activity and spread of this conjugative element. These interactions are reminiscent of abortive infection, in which bacterial cells possess a mechanism to kill themselves to prevent spread of an active phage [64]. An intriguing comparison is the abortive infection mechanism encoded by ICE*Bs1*, in which *spbK* (from ICE*Bs1*) protects host cells from predation by the co-resident prophage SPß [18]. However, in the case of Tn*916* and the defective prophage-like element *skin*, it is the prophage that appears to be limiting spread of the conjugative element. Following the abortive infection analogy, the ability of a host cell to limit spread of Tn*916* might protect neighboring cells from killing by Tn*916*. Alternatively, host cells may limit the spread of Tn*916* to limit the sharing of *tetM* and to possibly outcompete neighboring cells in the presence of tetracycline. Because Tn*916* is only activated in a small fraction of cells in a population, this model could be feasible. The interactions (direct or indirect) between the *yqaR* and *orf17*-*16* gene products represent a type of interaction and perhaps competition between mobile genetic elements that share a bacterial host.

### Multiple mechanisms by which Tn*916* causes growth arrest and cell death

Beyond the growth arrest and killing mediated by *yqaR* and *orf17*-*16*, Tn*916* has other genes that cause host cell death. In the absence of *yqaR*, there is growth arrest and cell death when Tn*916* is activated in *B*. *subtilis*. In addition, growth arrest and cell death also occur in *E*. *faecalis*, which has no recognizable homologs of *yqaR*. It is possible that there are functional analogs of YqaR, but we favor a model in which Tn*916* influences cell growth and viability through other pathways, both in its natural host *E*. *faecalis* and in *B*. *subtilis*. We suspect that similar processes occur in other natural hosts, and that close relatives of Tn*916* are likely to cause similar phenotypes.

### Cell fate and the spread of integrative and conjugative elements

A major question arising from our findings centers around the fate of the transconjugant cells that acquire a copy of Tn*916*. During conjugation, a linear single-stranded copy of Tn*916* is transferred from donor to recipient. Once in the recipient, the DNA re-circularizes and is replicated to form a dsDNA circle, which is the substrate for integration {reviewed in [3]}. The Tn*916* genes are presumably expressed from the dsDNA; in particular, the integrase needs to be made. Based on our results, we expect that expression of Tn*916* genes would be detrimental to the nascent transconjugants. However, it is clear that Tn*916* is able to successfully transfer and produce viable transconjugants, indicating that at least some fraction of nascent transconjugants are able to survive. Perhaps Tn*916* is able to integrate and thus halt expression of its detrimental genes in a short time scale that does not compromise the viability of its host cell. The initial acquisition of a conjugative element can be costly to host cell growth, and such a phenotype would not be unique to Tn*916*; previous reports have demonstrated the costs associated with conjugative element acquisition {e.g., [65–68]}. Future studies may explore the mechanisms of cell survival in transconjugants.

Similar questions apply to other ICEs. For example, ICE*clc* from *Pseudomonas* species is activated stochastically in 3–5% of cells in a population, and these cells differentiate into a “transfer competent” state that is characterized by slow growth, decreased viability, and the ability to transfer the element efficiently [9,69]. The genes required for the decreased cell growth and viability do not encode components of the conjugation machinery, but their loss causes a decrease in conjugation efficiency, indicating that the differentiated state is somehow important for efficient transfer of ICE*clc* [9]. This is in contrast to the situation with Tn*916*: at least some of the Tn*916* genes that contribute to the growth arrest and cell death encode proteins that are part of the conjugation machinery.

More similarly to Tn*916*, an essential component of the conjugation machinery encoded by the ICE R391, originally isolated from *Providencia rettgeri*, permeabilizes the host cell membrane, causing death [70,71]. This killing is proposed to function as a back-up mechanism for spread of the element through a population [58,72]. However, the mechanisms by which Tn*916* causes growth and viability defects appears to be different. First, the protein in R391 responsible for these phenotypes is not related to any of those encoded by Tn*916*. Second, for Tn*916*, partial alleviation of the growth and viability defects leads to an increase in transfer. Thus, for R391 and ICE*clc*, the growth arrest and decreased viability stimulate transfer whereas for Tn*916*, they inhibit transfer. These differences highlight how various ICEs and their hosts have evolved multiple and contrasting mechanisms that impact growth and viability of host cells and spread of the element.

We suspect that other ICEs have similarly complex impacts on their host cells. However, since most ICEs are activated in only a relatively small fraction of cells in a population, these effects are difficult to observe. The ability to activate an ICE in a large fraction of cells and to visualize and analyze individual cells that contain an active ICE should reveal many of the complex interactions that occur between an ICE, a host cell, and other horizontally acquired elements.

## Materials and methods

### Media and growth conditions

*B*. *subtilis* cells were grown shaking at 37°C in either LB medium or MOPS (morpholinepropanesulfonic acid)-buffered 1X S7_50_ defined minimal medium [73] containing 0.1% glutamate, required amino acids (40 μg/ml phenylalanine and 40 μg/ml tryptophan) and either glucose or arabinose (1% (w/v)) as a carbon source or on LB plates containing 1.5% agar.

*E*. *faecalis* cells were grown shaking at 37°C either in an M9 medium, consisting of 1X M9 salts supplemented with 0.3% yeast extract, 1% casamino acids, 3.6% glucose, 0.012% MgSO_4_, and 0.0011% CaCl_2_ [74,75] or in BHI medium. *Escherichia coli* cells were grown shaking at 37°C in LB medium for routine strain constructions. As appropriate, antibiotics were used in standard concentrations [76]: 5 μg/ml kanamycin, 12.5 μg/ml tetracycline, and 100 μg/ml streptomycin for selection on solid media.

### Strains, alleles, and plasmids

*E*. *coli* strain AG1111 (MC1061 F’ *lacI*^q^ *lacZ*M15 Tn*10*) was used for plasmid construction. *Bacillus subtilis* strains (Table 2), except BS49, were derived from JH642, contain the *trpC2 pheA1* alleles [77,78], and were made by natural transformation [76] or conjugation to introduce the indicated ICE. Key strains and newly reported alleles are summarized below.

**Table 2:** *B*. *subtilis* strains used.

Strain	Genotype^a^ (reference[s])
BS49	*metB5 hisA1 thr*-*5 att*(*yufKL*)::Tn*916 att*(*ykuC*-*ykyB*)::Tn*916* ICE*Bs1*^+^ [27,79,80]
CMJ253	*att*(*yufKL*)::Tn*916* [81]
JMA168	ICE*Bs1* [Δ(*rapI*-*phrI*)*342*::*kan*] *amyE*::[(Pspank(hy)-*rapI*) *spc*] [42]
JMA208	ICE*Bs1 immR*::*cat* (unstable and used to cure ICE*Bs1*) [42,81]
JMA222	ICE*Bs1*-cured [42] (ICE*Bs1*^0^)
LKM18	*att*(*nupQ*-*maeN*)::Tn*916*-*gfp* (ICE*Bs1*^0^)
LKM20	*att*(*ykuC*-*ykyB*)::Tn*916*-*gfp* (ICE*Bs1*^0^)
MMB970	ICE*Bs1* Δ(*rapI*-*phrI*)*342*::*kan amyE*::[(Pxyl-*rapI*) *spc*]
MO1100	*Δskin* [48,82] ICE*Bs1*^+^
ELC301	*comK*::*spc str*-*84* (ICE*Bs1*^0^)
ELC1076	ICE-H1-Δ*attR* Δ*orf20*^c^ {indicated as ICE-H1-*ΔattR* (Rep-)} *amyE*::[(Pxyl-*rapI*) *spc*]
ELC1095	ICE*Bs1*[Δ*nicK306* Δ(*rapI*-*phrI*)*342*::*kan ΔattR*::*tet*] *amyE*::[(Pxyl-*rapI*) *spc*]
ELC1213	(ICE*Bs1*-Tn*916*)-H1^b^ *amyE*::[(Pspank(hy)-*rapI*) *spc*]
ELC1214	(ICE*Bs1*-Tn*916*)-H1^b^ *amyE*::[(Pxyl-*rapI*) *spc*]
ELC1226	ICE*Bs1*[Δ(*helP*-*cwlT*) Δ(*yddJ*-*yddM*) *kan*] *amyE*::[(Pxyl-*rapI*) *spc*]
ELC1418	ICE-H1-Δ*attR* (Rep-)^c^ [Δ*orf15*] *amyE*::[(Pxyl-*rapI*) *spc*]
ELC1419	ICE-H1-Δ*attR* (Rep-)^c^ [Δ*orf17*] *amyE*::[(Pxyl-*rapI*) *spc*]
ELC1420	ICE-H1-Δ*attR* (Rep-)^c^ [Δ*orf16*] *amyE*::[(Pxyl-*rapI*) *spc*]
ELC1458	*att*(*yufKL*)::Tn*916*-*gfp*^d^ (ICE*Bs1*^0^)
ELC1491	*lacA*::[(P*xis*-*orf16*) *mls*]^e^ *cgeD*::[(P*immR*-(*immR*-*immA*) *kan*] *amyE*::[(Pxyl-*rapI*) *spc*] (ICE*Bs1*^0^)
ELC1494	*lacA*::[(P*xis*-*orf17*) *mls*]^e^ *cgeD*::[(P*immR*-(*immR*-*immA*) *kan*] *amyE*::[(Pxyl-*rapI*) *spc*] (ICE*Bs1*^0^)
ELC1495	*lacA*::[(P*xis*-empty) *mls*]^e^ *cgeD*::[(P*immR*-(*immR*-*immA*) *kan*] *amyE*::[(Pxyl-*rapI*) *spc*] (ICE*Bs1*^0^)
ELC1496	*lacA*::[(P*xis*-(*orf17*-*16*)) *mls*]^e^ *cgeD*::[(P*immR*-(*immR*-*immA*) *kan*] *amyE*::[(Pxyl-*rapI*) *spc*] (ICE*Bs1*^0^)
ELC1512	*att*(*yufKL*)::Tn*916*-*gfp Δorf17*-*16*^d^ (ICE*Bs1*^0^)
ELC1550	ICE-H1-Δ*attR* (Rep-)^c^ Δ*orf17*-*16 amyE*::[(Pxyl-*rapI*) *spc*] *lacA*::[(P*xis*-*orf17*-*16*) *mls*]^e^
ELC1705	ICE-H1-Δ*attR* (Rep-)^c^ Δ*orf13 amyE*::[(Pxyl-*rapI*) *spc*]
ELC1707	ICE-H1-Δ*attR* (Rep-)^c^ Δ*ardA amyE*::[(Pxyl-*rapI*) *spc*]
ELC1708	ICE-H1-Δ*attR* (Rep-)^c^ Δ*orf14 amyE*::[(Pxyl-*rapI*) *spc*]
ELC1760	*lacA*::[(P*xis*-(*orf17*-*16*)) *mls* (Pxyl-*rapI*) *spc*]^e^ *cgeD*::[(P*immR*-(*immR*-*immA*) *kan*] *amyE*::[(Pxyl-*rapI*) *cat* (P*immR*-(*immR*-*immA*)) (Pxis-(*orf17*-*16*))] *yhdGH*::[(P*xis*-*lacZ*) *tetM*] (ICE*Bs1*^0^)
ELC1830	Δ*skin* ICE*Bs1*-cured (ICE*Bs1*^0^) (MO1100 cured of ICE*Bs1*)
ELC1843	(ICE*Bs1*-Tn*916*)-H1^b^ *amyE*::[(Pspank(hy)-*rapI*) *spc*] Δ*yqaR*::*cat*
ELC1844	ICE*Bs1* Δ(*rapI*-*phrI*)*342*::*kan amyE*::[(Pspank(hy)-*rapI*) *spc*] Δ*yqaR*::*cat*
ELC1846	*att*(*yufKL*)::Tn*916 Δskin* (ICE*Bs1*^0^)
ELC1851	*att*(*yufKL*)::Tn*916* Δ*yqaR*::*cat* (ICE*Bs1*^0^)
ELC1854	*comK*::*spc str*-*84* Δ*yqaR*::*cat* (ICE*Bs1*^0^)
ELC1856	ICE-H1-Δ*attR* (Rep-)^c^ *amyE*::[(Pxyl-*rapI*) *spc*] Δ*yqaR*::*cat*
ELC1857	*att*(*yufKL*)::Tn*916*-*gfp* Δ*yqaR*::*cat* (ICE*Bs1*^0^)
ELC1891	*amyE*::[(Pxyl-*rapI*) *spc* (P*immR*-(*immR*-*immA*) (Pxis-(*orf17*-*16*))]^e^ Δ*skin* (ICE*Bs1*^0^)
ELC1892	*amyE*::[(Pxyl-*rapI*) *spc* (P*immR*-(*immR*-*immA*) (Pxis-(*orf17*-*16*))]^e^ Δ*yqaR*::*cat* (ICE*Bs1*^0^)
ELC1899	ICE-H1-Δ*attR* (Rep-)^c^ *orf16*(*K477E*) *amyE*::[(Pxyl-*rapI*) *spc*]
ELC1903	*amyE*::[(Pxyl-*rapI*) *spc* (P*immR*-(*immR*-*immA*) (Pxis-(*orf17*-*16*))]^e^ Δ*skin yhdGH*::[(P*yqaR*-*yqaR*) *kan*] (ICE*Bs1*^0^)
ELC1904	*amyE*::[(Pxyl-*rapI*) *spc* (P*immR*-(*immR*-*immA*) (Pxis-(*orf17*-*16*))]^e^ Δ*yqaR*::*cat yhdGH*::[(P*yqaR*-*yqaR*) *kan*] (ICE*Bs1*^0^)
ELC1908	ICE-H1-Δ*attR* (Rep-)^c^ *amyE*::[(Pxyl-*rapI*) *spc*] Δ*skin*
ELC1909	ICE-H1-Δ*attR* (Rep-)^c^ *amyE*::[(Pxyl-*rapI*) *spc*] Δ*skin yhdGH*::[(P*yqaR*-*yqaR*) *kan*]
ELC1911	ICE-H1-Δ*attR* (Rep-)^c^ *amyE*::[(Pxyl-*rapI*) *spc*] Δ*yqaR*::*cat yhdGH*::[(P*yqaR*-*yqaR*) *kan*]
ELC1915	ICE-H1-Δ*attR* (Rep-)^c^ Δ*orf19 amyE*::[(Pxyl-*rapI*) *spc*]
ELC1916	ICE-H1-Δ*attR* (Rep-)^c^ Δ*orf21 amyE*::[(Pxyl-*rapI*) *spc*]
ELC1918	*amyE*::[(Pxyl-*rapI*) *spc* (P*immR*-(*immR*-*immA*) (Pxis-(*orf17*-*16*))]^e^ Δ*yqaR*::*cat yhdGH*::*kan* (also indicated as Δ*yhdGH*::empty in contrast to ELC1922) (ICE*Bs1*^0^)
ELC1922	*att*(*yufKL*)::Tn*916 ΔyqaR yhdGH*::[(P*yqaR*-*yqaR*) *kan*] (ICE*Bs1*^0^)
ELC1923	*att*(*yufKL*)::Tn*916 Δskin yhdGH*::[(P*yqaR*-*yqaR*) *kan*] (ICE*Bs1*^0^)
ELC1942	ICE-H1-Δ*attR* (Rep-)^c^ Δ*orf17*-*16 amyE*::[(Pxyl-*rapI*) *spc*]
ELC1945	ICE-H1-Δ*attR* (Rep-)^c^ Δ*orf23*-*22 amyE*::[(Pxyl-*rapI*) *spc*]

^a^*B*. *subtilis* strains, except BS49, are derived from JH642 (AG174) and contain the *trpC2 pheA1* alleles (not written in the table) [77,78]. Genotypes indicate if a strain contains ICE*Bs1*, Tn*916*, or an ICE*Bs1-*Tn*916* hybrid (ICE-H1). Many strains are cured of ICE*Bs1*, indicated as ICE*Bs1*^0^. Original Tn*916* gene names (*orf1*-*24*) are used as appropriate.

^b^(ICE*Bs1*-Tn*916*)-H1 expanded genotype: ICE*Bs1*[Δ(*helP*-*yddM*)::(Tn*916*(*orf23*-*orf13*) *kan*)] [41].

^c^ICE-H1-Δ*attR* (Rep-) expanded genotype: ICE*Bs1*[Δ(*helP*-*yddM*)::(Tn*916*(*orf23*-*orf21*, *orf19*-*orf13*) *kan*) Δ*attR*::*tet*]. This is essentially (ICE*Bs1*-Tn*916*)-H1, containing the indicated genes from Tn*916*, except that *attR* and *orf20* are deleted. *orf20* encodes the relaxase needed for nicking and replication and the element is indicated as (Rep-).

^d^Tn*916*-*gfp* contains *gfpmut2* between *attL* and *orf24*.

^e^P*xis*-driven alleles use the P*xis* promoter from ICE*Bs1*, which is repressed by ImmR and activated by the metalloprotease ImmA and the cell-signaling receptor RapI [42,43,83].

Δ(*rapI*-*phrI*)*342*::*kan* was used to select for ICE*Bs1* during matings as described previously [42]. Δ*attR*::*tet* [44] was used to prevent element excision from the chromosome. ICE*Bs1* Δ*nicK* [84], and Tn*916* Δ*orf20* [32] prevent nicking and subsequent DNA unwinding of the cognate ICE and were previously described.

*B*. *subtilis* strains cured of ICE*Bs1* (ICE*Bs1*^0^) and the streptomycin resistance allele (*str*-*84*) were previously described [42]. Δ*comK*::*spc* in ELC301 replaced most of the *comK* open reading frame from 47 bp upstream of *comK* to 19 bp upstream of its stop codon with the spectinomycin resistance cassette from pUS19 [85]. The *spc* marker was fused with up- and downstream homology regions via isothermal assembly [86] and used for transformation.

The construction of (ICE*Bs1*-Tn*916*)-H1, a hybrid integrative and conjugative element, was previously described [41]. Essentially, we replaced the DNA processing and conjugation genes of ICE*Bs1* (*helP*-*cwlT*) with those of Tn*916* (*orf23-orf13*) (Fig 1). In this hybrid element, the Tn*916* genes are controlled by the promoter P*xis* from ICE*Bs1* (regulated by ImmR, ImmA, and RapI). The integration and excision components (Int and Xis) are from ICE*Bs1*. ICE-H1 is easily and efficiently activated by overproduction of the ICE*Bs1*-encoded activator protein RapI [41].

ICE*Bs1*, (ICE*Bs1*-Tn*916*)-H1, and complementation constructs were under the regulatory control of P*xis* (of ICE*Bs1*) and were activated by overexpression of *rapI* using either a xylose-inducible (*amyE*::[(Pxyl-*rapI*) *spc*]) [47] or an isopropyl-β-D-thiogalactopyranoside (IPTG)-inducible (*amyE*::[(Pspank(hy)-*rapI*) *spc*]) [42] copy of *rapI*. When needed, ICE-cured strains contained *cgeD*::[(P*immR*-(*immR*-*immA*) *kan*] [43] for regulation of P*xis* used to drive expression of various genes.

Tn*916* host strain CMJ253 contains a single copy of Tn*916* between *yufK* and *yufL* [81] (at GAAAGGGACT TTTTTATATG AAAAATACTT, where the underlined nucleotides indicate the Tn*916*-chromosome junction). It was generated by natural transformation of JMA222, a JH642-derived strain that was cured of ICE*Bs1* [42], with genomic DNA from BS49 [27,79,80], selecting for tetracycline resistance from Tn*916*, as previously described [32,81].

Unmarked deletions were generated for Tn*916* genes *orf23*-*orf13* in the context of the hybrid element ICE-H1. Briefly, flanking homology regions were amplified for each deletion and inserted by isothermal assembly into pCAL1422, a plasmid containing *E*. *coli lacZ* and *cat* in the backbone, cut with EcoRI and BamHI [87]. The resulting plasmids were used to transform an appropriate *B*. *subtilis* strain, selecting for integration of the plasmid into the chromosome (chloramphenicol resistant) by single crossover recombination. Transformants were screened for loss of *lacZ* and checked by PCR for the desired deletion. The deletion boundaries are described below.

Deletion of Tn*916* genes *orf23*-*22* (*Δorf23-22*) extends from immediately after the *orf24* stop codon through the *orf22* stop codon. *Δorf21* (encoding the coupling protein) removes the first 1272 bp of *orf21* and leaves the remaining 114 bp (and *oriT*) intact. Δ*orf19* extends from 5 bp upstream of *orf19* to 15 bp upstream of *ardA* (*orf18*), likely abolishing *sso916* which is between *orf19* and *ardA* [32]. Δ*ardA* removes most of the open reading frame, leaving the final 26 bp intact (to leave a previously misannotated *orf17* start codon intact).

Of note, we found that *orf17* actually begins 88 bp downstream of *ardA* (it was previously predicted to overlap with the last 26 bp of *ardA*; the actual start codon was previously predicted to be Met39). The misannotated start site lacked an obvious ribosome binding site; we found that an ectopic expression allele using the *orf17* “downstream” start site was able to restore mating for a donor strain containing (ICE*Bs1*-Tn*916*)-H1(Δ*orf17*), which could not detectably mate.

Δ*of17* extends from 78 bp upstream of the *orf17* start codon and leaves the last 14 codons intact. Δ*orf16* removes codons 10–804 (of 815 total). Δ*orf15* removes codons 6–716 (of 754 total), based on the sequence for Tn*916* in *Enterococcus faecalis* DS16 (GenBank U09422.1). However, *orf15* in Tn*916* from BS49 contains a cytosine insertion resulting in a 725-amino acid protein [79]. This frameshift was removed in the deletion, allowing codons 1–5 to be fused with the originally annotated codons 716–754. Δ*orf14* removes codons 32–325 (of 333 total).

Ectopic expression alleles controlled by P*xis* to test the sufficiency of Tn*916* genes to cause growth defects were cloned at *lacA*, as previously described [7]. Briefly, *orf16*, *orf17*, or *orf17*-*16* together were fused to P*xis*, an MLS-resistance cassette (with its own promoter), and up- and downstream homology arms were combined via isothermal assembly and transformed into *B*. *subtilis*, selecting for acquisition of MLS resistance. A *lacA*::[(P*xis*-empty) *mls*] with no gene downstream of Pxis, was constructed similarly and was used as a control.

ELC1760 was used to screen for suppressors of the cell death caused by expression of *orf17*-*16*. The screen was done in a strain with two copies of *orf17*-*16* and two copies of each of the genes required for regulation (*immR*, *immA*, and *rapI*). Having two copies of each gene virtually eliminated suppressor mutations related to *orf17*-*16* expression because the frequency of mutations in both copies of a duplicated gene would be exceedingly low. Previous constructs were used to generate additional alleles. *lacA*::[(P*xis*-*orf17*-*16*) *mls* (Pxyl-*rapI*) *spc*] was constructed by inserting [(Pxyl-*rapI*) *spc*] into the existing *lacA*::[(P*xis*-*orf17*-*16*) *mls*] allele, selecting for spectinomycin resistance. *amyE*::[(*Pxyl*-*rapI*) *cat* (*PimmR*-(*immR*-*immA*) (*Pxis*-(*orf17*-*16*))] was constructed by inserting *cat*, *PimmR*-(*immR*-*immA*), and *Pxis*-(*orf17*-*16*) into *amyE*::[(*Pxyl*-*rapI*) *spc*] and selecting for chloramphenicol resistance.

*yhdGH*::[(P*xis*-*lacZ*) *tetM*] was generated by fusing P*xis*, *lacZ*, and *tetM*, with *yhdG* and *yhdH* homology arms via isothermal assembly, transforming into *B*. *subtilis*, and selecting for tetracycline resistance.

Δ*yqaR*::*cat* is a deletion-insertion replacing *yqaR* from 18 bp upstream of the start of the *yqaR* open reading frame to 48 bp upstream of its stop codon with the chloramphenicol resistance cassette from pGEMcat [88]. Fragments were joined via isothermal assembly and used for transformation. A *B*. *subtilis* strain cured of the *skin* element (leaving behind intact *sigK*) was obtained from the Losick lab {*Δskin* allele described in [48,82]}.

*yhdGH*::[(P*yqaR*-*yqaR*) *kan*] is an insertion of *yqaR*, from 275 bp upstream of the open reading frame through its stop codon and apparently containing its native promoter. *yqaR* and *kan* were cloned between *yhdG* (*bcaP*) and *yhdH*. The construct was inserted 19 bp downstream from the stop codon of *yhdG* and 98 bp upstream of the *yhdH* start codon such that *yqaR* and *kan* are in the opposite orientation of *yhdG* and *yhdH*. The transcriptional terminator from between *serA* and *aroC* (from 2 bp downstream of the *serA* stop codon to 6 bp downstream of the convergent *aroC* stop codon) was amplified from the *B*. *subtilis* chromosome and inserted upstream of *yqaR* to insulate it from possible transcription from upstream sequences.

Tn*916*-*gfp* was generated as a reporter to monitor Tn*916* activation in single cells. It is an unmarked insertion of promoterless *gfpmut2* 29 bp upstream of *orf24*. *gfpmut2* was cloned into pCAL1422 (described above) to generate pELC1329. This plasmid was used to generate *B*. *subtilis* strain ELC1458, which contains a copy of Tn*916*-*gfp* between *yufL* and *yufK*. In this context, *gfpmut2* (along with the rest of the DNA processing and T4SS genes) will not be expressed until the element has excised from the chromosome and circularized [33]. To confirm that the growth defects observed upon activation of Tn*916*-*gfp* in ELC1458 were not due to this particular integration site of Tn*916*, this element was subsequently mated into JMA222 (which lacks Tn*916* and ICE*Bs1*) to isolate strains LKM18 and LKM20, which contained Tn*916*-*gfp* at different chromosomal sites: between *nupQ*-*maeN* (TTAGTTTTTT AACTTAAAAA AATATGAAGT) and between *ykuC*-*ykyB* (CAGGTTAAAA ATGCGCTTTT TTTCTTAGAA), respectively. New integration sites were mapped by arbitrary PCR, as previously described [41,89,90].

Tn*916*-*gfp* was transferred via conjugation from *B*. *subtilis* donors into *E*. *faecalis* (ATCC 19433) recipients under standard mating conditions (described below). Briefly, *B*. *subtilis* Tn*916-gfp* donors were D-alanine auxotrophs (Δ*alr*::*cat*) and the absence of D-alanine was used as a counter-selection against donors when selecting for *E*. *faecalis* Tn*916*-*gfp* transconjugants [41,89]. To transfer Tn*916* (Δ*orf17*-*orf16*) into *E*. *faecalis*, the *Δorf17-16* deletion mutation was complemented with a copy of *orf17-16* elsewhere in the donor genome.

Tn*916* insertion sites were identified by arbitrary PCR or inverse PCR, similar to previously described methods [32,44]. Briefly, for inverse PCR, chromosomal DNA was digested with either PacI or AseI/NdeI restriction enzymes and then ligated to circularize the fragments. The following primer pairs were used to amplify and sequence the Tn*916*-chromosome junctions: oLM177 (5’- AACGCTTCGT TATGTACCCT CTG) and oLM178 (5’–ACCACTTCTG ACAGCTAAGA CATG) for PacI digested DNA; oLW443 (5’–CTCTACGTCG TGAAGTGAGA ATCC) and oLW209 (5’–TTGACCTTGA TAAAGTGTGA TAAGTCC) for AseI/NdeI digested DNA. Integration sites were mapped to the following chromosomal regions of the *E*. *faecalis* genome (Accession number: ASDA00000000.1).

We made two different *E*. *faecalis* strains containing Tn*916-gfp* and one that contained Tn*916-gfp Δ*(*orf17-orf16*) and determined the site of each insertion. The location of each insertion was based on the sequence annotation of *E*. *faecalis* KB1, accession number: CP015410.1. For genomic context, thirty bases of sequence are shown, and the underlined nucleotides indicate the predicted Tn*916*-chromosome junction.

ELC1529 has two copies of Tn*916*. One was between *citG* and a gene encoding a surface protein (SP), *att*(*citG_*SP)::Tn*916*-*gfp*: (AACGGCTGTC GCCTTTTTTT ATGAAATTTT). The second was between genes encoding a hypothetical protein (HP) and a cold shock protein (CSP), *att*(HP_CSP)::Tn*916*-*gfp*: (TTTCTTGTTC TTTTTTTTAT AAAAAAAACC).

ELC1531 has a copy of Tn*916* between genes encoding a predicted ABC transporter (ABC) and an acyl carrier protein (ACP), *att*(ABC_ACP)::Tn*916*-*gfp*: (TTTTTTACAT GTATGATTTT TTTTACAAAA).

ELC1696 contained Tn*916* Δ*orf17*-*orf16* between genes encoding an alpha/beta hydrolase (ABH) and a hypothetical protein, *att*(ABH_HP)::Tn*916*-*gfp* Δ(*orf17*-*orf16*): (TCTTTTTTTT GTAATAAAAA ACAGAAAATT).

### Growth and viability assays

*B*. *subtilis* strains were grown in defined minimal medium with 1% arabinose as a carbon source to early exponential phase. At an OD_600_ of 0.2, the cultures were split into activating or non-activating conditions: 1% xylose was added to stimulate activation of ICE*Bs1*, (ICE*Bs1*-Tn*916*)-H1, or ectopic expression constructs; 2.5 μg/ml tetracycline was added to stimulate activation of Tn*916*. The number of colony forming units (CFUs) was determined immediately prior to activation, and at one or more time points (typically three hours) after activation in induced and non-induced cultures. “Relative viability” was calculated as the number of CFUs present in the induced culture divided by the number of CFUs present in the non-induced culture. OD_600_ was monitored every 30 minutes for four hours post-induction.

### Excision assays

qPCR was used to monitor excision (and therefore activation) of Tn*916*, ICE-H1, and ICE*Bs1*, as previously described [32,41]. Briefly, gDNA of ICE host strains was harvested using the Qiagen DNeasy kit with 40 mg/ml lysozyme. The primers described below were used to quantify the presence of the empty ICE attachment site, normalized to a nearby chromosomal locus that is present in every cell.

For Tn*916* excision assays (integrated between *yufK* and *yufL*), we used previously described primers [32]: oLW542 (5’- GCAATGCGAT TAATACAACG ATAC) and oLW543 (5’- TCGAGCATTC CATCATACAT TC) amplified the empty chromosomal attachment site (*att1*). oLW544 (5’- CCTGCTTGGG ATTCTCTTTA TC) and oLW545 (5’- GTCATCTTGC ACACTTCTCT C) amplified a region within the nearby gene *mrpG*.

For ICE-H1 and ICE*Bs1* (integrated at *trnS*-*leu2*), oMA198 (5’- GCCTACTAAA CCAGCACAAC) and oMA199 (5’- AAGGTGGTTA AACCCTTGG) amplified the empty chromosomal attachment site (*attB*). oMA200 (5’- GCAAGCGATC ACATAAGGTT C) and oMA201 (5’- AGCGGAAATT GCTGCAAAG) amplified a region within the nearby gene, *yddN*.

qPCR was performed using SsoAdvanced SYBR master mix and the CFX96 Touch Real-Time PCR system (Bio-Rad). Excision frequencies were calculated as the number of copies of the empty chromosomal attachment sites (as indicated by the Cp values measured through qPCR) divided by the number of copies of the nearby chromosomal locus. Standard curves for these qPCRs were generated using *B*. *subtilis* genomic DNA that contained empty ICE attachment sites and a copy of the nearby gene (*yddN* or *mrpG*). DNA for the standard curves was harvested when these strains were in late stationary phase and had an *oriC*/*terC* ratio of ~1, indicating that the copy numbers of these targets were in ~1:1 ratios.

### Suppressor screen

Eighteen independent cultures of ELC1760, which contains two copies of *orf17* and *orf16* (described above, Table 2) were grown in defined minimal medium (with 1% arabinose). In early exponential phase, expression of *orf17* and *orf16* was induced with 1% xylose and cultures were grown overnight (approximately 18 hours) until they became dense. Cultures were back-diluted and this process was repeated once to enrich for suppressor mutants. Some cultures were back-diluted a second time to further enrich and increase the chances of isolating suppressors. Suppressors were colony-purified, and checked for presence of all antibiotic resistance markers. Additionally, we confirmed these isolates properly activated P*xis*-*lacZ* when streaked on LB plates containing X-gal (5-bromo-4-chloro-3-indolyl-β-D-galactopyranoside) and 1% xylose, indicating that the RapI-driven induction of P*xis* was still working properly (and likely *orf17* and *orf16* were still being expressed).

### Genome resequencing

Each suppressor mutant was grown in a minimal medium containing 1% glucose to an OD_600_ ~1.5–2. Cells were harvested, and DNA was extracted using a Qiagen 100 G tips purification kit. Sample preparation, including DNA shearing using a Covaris ultrasonicator, size selection (aiming for ~500 bp), adapter ligation, and paired-end read sequencing (300nt + 300nt) on an Illumina MiSeq were performed by the MIT BioMicro Center. Reads were mapped to the *B*. *subtilis* JH642 chromosome (Accession number: CP007800) [78], as previously described [91].

### Mating assays

Mating assays were performed essentially as described previously [42]. Briefly, donor strains containing Tn*916* (tetracycline-resistant), (ICE*Bs1*-Tn*916*)-H1 (kanamycin-resistant), or ICE*Bs1* {Δ(*rapI-phrI*)::*kan*}, or derivatives were grown in LB medium to early exponential phase. At an OD_600_ ~0.2, Tn*916* activation was stimulated with 2.5 μg/ml tetracycline; ICE-H1 and ICE*Bs1* activation was stimulated with 1mM IPTG (via the P*spank*(*hy*)-*rapI* allele). After one hour induction, donor strains were mixed in a 1:1 ratio with streptomycin resistant recipient cells (5 total ODs of cells) and filtered. Mating filters were placed on a 1X Spizizen’s salts [76] 1.5% agar plate at 37°C for one hour. Cells were then harvested off the filter and plated on selective media to detect ICE transfer. The number of donor (tetracycline or kanamycin resistant), recipient (streptomycin resistant), and transconjugant (tetracycline/streptomycin resistant or kanamycin/streptomycin resistant) CFUs were determined both pre- and post-mating. Conjugation efficiency was calculated as the percentage of transconjugant CFUs per donor cell at the start of mating. Conjugation efficiencies were normalized to those of the wild type matings performed in parallel. Typically, conjugation efficiencies were as follows: Tn*916* ~0.0005%, ICE-H1 ~0.5%, ICE*Bs1* ~1%.

### Time-lapse microscopy and analysis

*B*. *subtilis* and *E*. *faecalis* cells were grown to early-exponential phase in the appropriate medium. When applicable, Tn*916* activation was stimulated with 2.5 μg/ml tetracycline. After a 1–3 hour induction, cells were transferred to an agarose pad (1.5% UltraPure agarose; Invitrogen) containing growth medium, 0.1 μM Propidium iodide, DAPI (0.035 μg/ml for *B*. *subtilis*; 0.5 μg/ml for *E*. *faecalis*), and either 2.5 μg/ml tetracycline for Tn*916* activation or 1% xylose for expression of *orf17*-*orf16*-*gfp*. The agarose pad was placed in an incubation chamber, which was made by stacking two Frame-Seal Slide Chambers (Bio-Rad) on a standard microscope slide (VWR). Cells were then grown at 37°C for 2–4 hours while monitoring growth. Time-lapse images were captured on a Nikon Ti-E inverted microscope using a CoolSnap HQ camera (Photometrics) using a Nikon Intensilight mercury illuminator and appropriate sets of excitation and emission filters (Chroma; filter sets 49000, 49002, and 49008). ImageJ software was used for image processing.

## Supporting information

S1 Video Growth arrest and death of *B*. *subtilis* cells with an activated Tn*916*. Cells containing Tn*916*-*gfp* integrated in the *B*. *subtilis* chromosome between *yufK*-*yufL* (ELC1458) were grown in defined minimal glucose medium to late exponential phase with 2.5 μg/ml tetracycline to stimulate Tn*916* excision. At time = 0 h, cells were spotted on minimal glucose agarose pads containing 2.5 μg/ml tetracycline, 0.1μM propidium iodide, and 0.035 μg/ml DAPI. Cells were monitored by phase contrast and fluorescence microscopy for three hours. A representative video from these experiments that highlights a single Tn*916*-activated cell undergoing lysis is shown here. GFP (green) was produced in cells in which Tn*916* was activated and excised from the chromosome. Propidium iodide (red) indicates dead cells.
(AVI)

S2 Video Growth arrest and death of *B*. *subtilis* cells with an activated Tn*916*. As in S1 video, cells containing Tn*916*-*gfp* integrated in the *B*. *subtilis* chromosome between *yufK*-*yufL* (ELC1458) were grown in defined minimal glucose medium to late exponential phase with 2.5 μg/ml tetracycline to stimulate Tn*916* excision. At time = 0 h, cells were spotted on minimal glucose agarose pads containing 2.5 μg/ml tetracycline, 0.1μM propidium iodide, and 0.035 μg/ml DAPI. Cells were monitored by phase contrast and fluorescence microscopy for three hours. A representative video from these experiments highlighting several Tn*916*-activated cells exhibiting growth defects and undergoing lysis is shown here. GFP (green) was produced in cells in which Tn*916* was activated and excised from the chromosome. Propidium iodide (red) indicates dead cells.
(AVI)

S3 Video Growth arrest and death of *E*. *faecalis* cells with an activated Tn*916*. *E*. *faecalis* cells containing two copies of Tn*916*-*gfp* (ELC1529) were used to monitor effects of Tn*916* activation. Cells were grown in a rich M9 medium (Methods) to late exponential phase with 2.5 μg/ml tetracycline to stimulate Tn*916* excision. At time = 0 h, cells were spotted on rich M9 medium agarose pads containing 2.5 μg/ml tetracycline, 0.1 μM propidium iodide, and 0.5 μg/ml DAPI. Cells were monitored by phase contrast and fluorescence microscopy for two hours. A representative video monitoring ELC129 cells with activated Tn*916*-*gfp* (GFP-positive) is shown here. The black arrow in the final frame indicates a PI-stained, GFP-positive cell (appears red or reddish-yellow).
(AVI)

S1 Data Underlying raw data for experiments presented in the figures. The excel spreadsheet contains the underlying data for the experiments presented in each of the figures.
(XLSX)
